# Rewiring of purine metabolism in response to acidosis stress in glioma stem cells

**DOI:** 10.1038/s41419-021-03543-9

**Published:** 2021-03-15

**Authors:** Xiaoyu Xu, Liping Wang, Qingce Zang, Shanshan Li, Limei Li, Zhixing Wang, Jiuming He, Boqin Qiang, Wei Han, Ruiping Zhang, Xiaozhong Peng, Zeper Abliz

**Affiliations:** 1 State Key Laboratory of Bioactive Substance and Function of Natural Medicines, Institute of Materia Medica, Chinese Academy of Medical Sciences and Peking Union Medical College, Beijing, China; 2 State Key Laboratory of Medical Molecular Biology, Department of Molecular Biology and Biochemistry, Institute of Basic Medical Sciences, Biomedical Primate Research Center, Neuroscience Center Chinese Academy of Medical Sciences, School of Basic Medicine Peking Union Medical College, Beijing, China; 3 Institute of Medical Biology, Chinese Academy of Medical Sciences and Peking Union Medical College, Kunming, China; 4 Centre for Bioimaging and Systems Biology, Minzu University of China, Beijing, China

**Keywords:** Glioma stem cells, Metabolomics, Acidic microenvironment, Glucose-6-phosphate dehydrogenase, Purine metabolism, Cancer metabolism, Cancer microenvironment

## Abstract

Glioma stem cells (GSCs) contribute to therapy resistance and poor outcomes for glioma patients. A significant feature of GSCs is their ability to grow in an acidic microenvironment. However, the mechanism underlying the rewiring of their metabolism in low pH remains elusive. Here, using metabolomics and metabolic flux approaches, we cultured GSCs at pH 6.8 and pH 7.4 and found that cells cultured in low pH exhibited increased de novo purine nucleotide biosynthesis activity. The overexpression of glucose-6-phosphate dehydrogenase, encoded by *G6PD* or *H6PD*, supports the metabolic dependency of GSCs on nucleotides when cultured under acidic conditions, by enhancing the pentose phosphate pathway (PPP). The high level of reduced glutathione (GSH) under acidic conditions also causes demand for the PPP to provide NADPH. Taken together, upregulation of G6PD/H6PD in the PPP plays an important role in acidic-driven purine metabolic reprogramming and confers a predilection toward glioma progression. Our findings indicate that targeting G6PD/H6PD, which are closely related to glioma patient survival, may serve as a promising therapeutic target for improved glioblastoma therapeutics. An integrated metabolomics and metabolic flux analysis, as well as considering microenvironment and cancer stem cells, provide a precise insight into understanding cancer metabolic reprogramming.

## Introduction

Metabolic alteration and rewiring are important hallmarks of cancer^1–5^. To adapt to the microenvironment of hypoxia, low pH, or nutrient deficiency, neoplastic cells reprogram multiple pathways regulating energy metabolism and biosynthetic metabolism in order to meet the increasing demands of growth and proliferation. As cancer cells are dependent on metabolic reprogramming, the key metabolic enzymes of these altered pathways hold immense potential as therapeutic targets^6–9^.

Glioblastoma (World Health Organization grade IV glioma) is the most common and lethal malignant primary brain tumor, with high recurrence and poor prognosis^10,11^. One of the most difficult to treat solid tumors, it is vital to consider tumor heterogeneity and variations in the metabolism of subpopulations of glioma cells to understand the resistance and recurrence of glioblastoma. One tumorigenic subpopulation, glioma stem cells (GSCs), has been shown to be tightly related to the resistance to radiation and chemotherapy of glioma^12,13^. The plasticity of cancer stem cells that enables them to adapt to distinct metabolic conditions and demands is considered an important hallmark in cancer development^1,14^. GSCs have the ability to repopulate tumors and promote secondary recurrence post-treatment^15,16^. Compared to differentiated tumor cells, GSCs maintain a distinct metabolic phenotype. Therefore, recent studies of glioma metabolism have advanced past the stage of parsing the metabolic disparity between tumor and normal cells and have begun to examine the specific metabolic changes of GSCs. In contrast to the primary metabolic profile across gliomas, it has been demonstrated that GSC metabolism can transform between oxidative phosphorylation (OXPHOS) and glycolysis depending on microenvironment^17^. Wang et al.^18^ showed that brain tumor-initiating cells in glioma preferentially demand de novo purine synthesis to maintain self-renewal, proliferation, and stemness. In addition, we previously reported on the association of significantly altered metabolites with the self-renewal and differentiation of GSCs (ref. ^19^). These studies underscore the metabolic heterogeneity of GSCs and the importance of their metabolic characteristics.

Past research has indicated that GSCs are maintained in specific microenvironments and are especially enriched in hypoxic and acidic areas, perinecrotic compartments, and perivascular niches^20,21^. GSCs show a dynamic interaction with their microenvironment with constant bidirectional cross-talk^14^, which is essential for glioblastoma initiation, proliferation, invasion, recurrence, and therapeutic resistance^22^. A main feature of the glioma microenvironment is low pH, of ~6.8 (refs. ^23,24^). Emerging evidence suggests that an acidic microenvironment promotes a GSC phenotype^23,25,26^. The ability to regulate GSCs metabolism in response to acidosis may suggest an important aspect to the resistance phenotype that these stem cells perform. Therefore, a greater understanding of the mechanism by which GSCs rewire their metabolic pathways to adapt to acidic conditions may yield new therapeutic targets for blocking the pH-regulatory systems of GSCs.

Recent studies have revealed the modulation of cancer cells under acidic stress. Lactate, a by-product of the Warburg effect, is a key metabolite in the acidic microenvironment of cancer cells. The initial adaption of cancer cell metabolism to an acidic microenvironment is related to lactate, which plays an important role in the epigenetic modification of tumors^27^. Mitochondrial reprogramming is another important alteration often found in tumor cells residing in an acidic microenvironment. Acidosis can override oxygen deprivation to maintain cell survival and mitochondrial function^28^. The results from our prior studies indicate that acidosis also enhances the self-renewal and mitochondrial respiration of GSCs through CYP24A1-mediated reduction of vitamin D^29^. Lipid and glutamine metabolic reprogramming in response to acidosis may also be a common mechanism in cancer cells. Corbet et al.^30^ reported that acidosis profoundly induced metabolic rewiring of cervical cancer cells toward fatty acid oxidation through alterations in mitochondrial and histone acetylation. Another study revealed that chronic acidosis stress in pancreatic cancer cells enhanced anaplerotic glutamine metabolism through the increased expression of the transaminase enzyme GOT1 (ref. ^31^).

Despite considerable interest in targeting metabolic pathways in GSCs, few studies have focused on the metabolic response to acidosis of GSCs. The combination of metabolomics and metabolic flux analysis can provide comprehensive information, whether static metabolome or dynamic flux changes. We employed this integrative analysis approach to characterize the metabolism of GSCs under low pH conditions and provide insight into metabolic reprogramming mechanism of GSCs in response to acidic stress. Further biological validation revealed potential therapeutic targets in dysregulated metabolic pathways of GSCs residing within an acidic microenvironment.

## Materials and methods

### Cell lines, culture conditions and treatments, and chemicals

The isolation, culture, and identification of GSC2 cells were performed as described previously^32^. Human GSC lines (T12-1 and T2-4) were obtained from Beijing Tiantan Hospital of Capital Medical University, Beijing, China. The GSCs were cultured in Neurobasal Medium (Gibco, Carlsbad, CA, USA) supplemented with 2% B27 (without vitamin A, Gibco, Carlsbad, CA, USA), 10-mg/mL heparin (Sigma, St. Louis, MO, USA), 20-ng/mL bFGF (Peprotech, Rocky Hill, NJ, USA), 20-ng/mL EGF (Peprotech, Rocky Hill, NJ, USA), 1% penicillin/streptomycin (Gibco, Carlsbad, CA, USA), and 1% glutamine (Gibco, Carlsbad, CA, USA). The human glioblastoma cell line LN229 was purchased from the American Type Culture Collection (ATCC) and cultured in Dulbecco’s modified Eagle’s medium, which was supplemented with 10% fetal bovine serum (HyClone, Logan City, UT), 100-U/mL penicillin (Life Science), and 100-U/mL streptomycin (Life Science). The cells were maintained at 37 °C in a humidified atmosphere containing 5% CO_2_–95% air.

For the pH treatment of cells, 1-M HCl and 1-M NaOH were used to adjust the pH values to 6.8 and 7.4 in medium supplemented with 25-mM HEPES (Sigma, USA). To maintain the appropriate pH, the medium was re-titrated after 24 h.

For isotope tracing analysis, Neurobasal Medium (Gibco, Carlsbad, CA, USA) without glucose and glutamine was supplemented with 25-mM ^13^C_6_-glucose or 2-mM ^15^N_2_-glutamine (Cambridge Isotope Laboratories, Tewksbury, MA) and then adjusted to the target pH as described above. GSCs were cultured in medium without tracers for ~12 h. The culture medium was then replaced with medium containing a tracer. After 36 h, the cells were prepared for LC–MS analysis. The cells were labeled for different times to determine the stable labeling time.

For addition of nucleotide metabolites to medium, three different concentration gradients of AMP (Sigma, 01930), GMP (Sigma, G8377), IMP (Sigma, I4625), and XMP (CAYMAN, 18134) were configured with normal saline such that the final concentrations of 0.1, 0.5, and 1 mM were added to the medium. In the control group, the cells were cultured normally with the same volume of saline without metabolites.

### Sample preparation for LC–MS analysis

Metabolites from GSCs were extracted using 80% methanol with three freeze-thaw cycles, according to our previous protocol^33^. GSCs were grown in suspension and were pelleted by centrifugation (1000 rpm, 25 °C, 5 min) before the first step in our protocol. For metabolomics analysis, dried samples were resuspended in 150 μL of 98% aqueous acetonitrile (initial mobile phase gradient), whereas for metabolic flux analysis, the dried samples were resuspended in 150 μL of 50% aqueous acetonitrile (for used in HILIC mode).

### LC–MS analysis

#### Untargeted metabolomics analysis

Untargeted metabolomics analysis was conducted on a Q-Exactive mass spectrometer with a heated electrospray ionization source (Thermo Fisher Scientific, Waltham, MA, USA), coupled to a Dionex UHPLC Ultimate 3000 system (Thermo Scientific, Dionex, Sunnyvale, California, USA).

Each sample in a volume of 10 μl was injected onto a Waters HSS T3 column (100 × 2.1 mm, 1.8 µm) for chromatographic separation. Mobile phases A and B were H_2_O with 0.1% formic acid and 100% acetonitrile, respectively. Gradient elution was performed using a flow rate of 0.25 mL/min as follows: 0 min, 2% B; 9 min, 60% B; 18 min, 60% B; 20 min, 100% B; and 30 min, 100% B. The column and autosampler temperatures were set at 35 and 4 °C, respectively.

Data were acquired using Xcalibur version 3.0 software (Thermo Fisher Scientific) in both positive and negative ion modes for a full scan with a mass range from 100 to 1000 m/z. Mass spectrometric parameters were set as follows: spray voltage, 3.5 kV for positive ion mode and −3.2 kV for negative ion mode; sheath gas flow rate, 40 arbitrary units (arb) for positive ion mode and 45 arb for negative ion mode; auxiliary gas, 11 arb for positive ion mode and 10 arb for negative ion mode; capillary temperature, 350 °C; S-lens RF level, 55; resolution, 70000; automatic gain control target, 3e6; and maximum injection time, 100 ms. A quality control sample containing equal aliquots of all cell samples was analyzed at eight sample intervals to monitor the stability of the LC–MS system. The injection sequence of cell samples was randomized to avoid machine drift.

#### Targeted metabolomics analysis

Targeted metabolomics analysis was performed on a UPLC system (Waters, Milford, MA, USA) coupled to a QTRAP 5500 mass spectrometer (Applied Biosystems SCIEX, Foster City, CA, USA).

Chromatographic separation was performed on a Synergi Hydro-RP column (2.0 × 250 mm, 4 μm) (Phenomenex, Torrance, CA, USA), and the column temperature was maintained at 35 °C. The mobile phase was prepared as described in untargeted metabolomics experiment. At a flow rate of 0.25 mL/min, the gradient conditions were as follows: 0 min, 2% B; 5 min, 2% B; 20 min, 100% B; and 25 min, 100% B. The autosampler temperature was maintained at 4 °C and the injection volume was 5 μL.

Multiple-reaction monitoring experiments were performed in both positive and negative ESI modes, and the data were acquired with Analyst version 1.6.1 software (Applied Biosystems SCIEX). The mass spectrometer parameters for each analyte including declustering potentials, collision energies, and suitable product ions were optimized and are shown in Supplementary Tables S1 and S2. Other detailed mass spectrometry parameters were set as follows: ESI source voltage, 5.5 or −4.5 kV; dwell time, 10 ms; source temperature (TEM), 450 °C; nebulizer gas, 70 psi; turbo gas, 60 psi; curtain gas, 30 psi; and collision-activated dissociation gas level, medium. L-Tryptophan-(indole-d_5_) (10 μg/mL), Cholic acid-2,2,3,4,4-d_5_ (10 μg/mL), L-Tyrsoine-^13^C_9_,^15^N (2 μg/mL), Palmitoyl-1-^13^C-L-carnitine hydrochloride (5 μg/mL), and L-Proline-^15^N (4 μg/mL) were chosen as internal standards to monitor the stability of the system.

#### Metabolic flux analysis

Metabolic flux analysis was performed on a 6550 Q-TOF mass spectrometer with Dual AJS ESI source (Agilent Corporation, MA, USA) equipped with a 1260 RRLC system (Agilent Co.).

LC separation was conducted under both acidic and basic conditions using a Waters BEH amide column (2.1 × 100 mm, 1.7 μm) at a column temperature of 35 °C. Mobile phase A was 95:5 acetonitrile: water with 10-mM ammonium acetate. Mobile phase B was H_2_O with 10-mM ammonium acetate. Ammonium hydroxide (0.05%) and acetic acid (0.05%) were added in basic and acidic LC conditions, respectively.

The injection volume was 5 μL, and the flow rate was 0.25 mL/min. The basic gradient conditions were 0 min, 15% B; 12 min, 40% B; and 15 min, 47% B. The acidic gradient conditions were 0 min, 5% B; 5 min, 30% B; 6 min, 50% B; and 12 min, 50% B. The MS parameters were set as follows: Gas Temp, 200 °C; Drying Gas, 14 l/min; Nebulizer: 35 psig; sheath gas temp, 350 °C; sheath gas flow: 11 l/min; VCap, 3500 V; Nozzle Voltage, 600 V; Fragmentor, 380 V; and Octopole RF, 750 V. The data were acquired in a mass range from 70 to 1000 *m/z* in negative ion mode using MassHunter workstation (Agilent Co., MA, USA).

### LC–MS data processing and data analysis

For global metabolomics, the raw data files were converted to the mzXML format using MassMatrix file conversion tools (http://mm-file-conversion.software.informer.com/3.9/). Then, the data were preprocessed for peak finding, filtering, alignment, matching, and identification in R software (version 2.15.2; R project, Vienna, Austria) by loading the R package of XCMS (ref. ^34^). The peak area for each detected feature was normalized by the total ion intensity of the sample. The data matrices were mean-centered and pareto-scaled and then imported into SIMCA-P 14.0 (Umetrics, Sweden) for principal component analysis and orthogonal partial least squares discriminant analysis (OPLS-DA). For targeted metabolomics analysis, chromatographic peak integration was performed with MultiQuant version 3.0.2 software (Applied Biosystems SCIEX). For metabolic flux analysis, MassHunter VistaFlux (Agilent Co., MA, USA) was used to process the data and correct isotopic natural abundance.

Pathway analysis of the differential metabolites was done using MetaboAnalyst 3.0 (http://www.MetaboAnalyst.ca/)^35^. A pathway with an impact factor >0.1 was considered an important pathway.

The MS spectra, MS/MS spectra, and retention time were used for metabolite identification by comparing them with the data of authentic standards. Human Metabolome Database (ref. ^36^) (http://www.hmdb.ca/) and Metlin^37^ (http://metlin.scripps.edu/) were also used for metabolites searching and validation.

### Statistical analysis

The experimental data are presented as the means ± SD. The statistical analysis was performed using a two-tailed Student’s *t*-test. *p* < 0.05 was considered statistically significant. In the figures, asterisks denote statistical significance between two groups (*p < 0.05; **p < 0.01; ***p < 0.001).

### Western blotting

Cells were digested with Accutase (Gibco, Carlsbad, CA, USA) and lysed in TNTE buffer with protease inhibitors (Sigma, St. Louis, MO, USA). Subsequently, the lysates were subjected to SDS-PAGE, and the proteins were transferred to nitrocellulose membranes. The following primary antibodies were used: anti-Nestin (1:500, ABclonal, A11861), anti-SOX2 (1:3000, Cell Signaling Technology, 5067S), anti-H6PD (1:500, Sigma, A06407), anti-IMPDH1 (1:1000, Abcam, ab33039), anti-PPAT (1:500, ORIGENE, TA504559), anti-G6PD (1:1000, Hangzhou HuaAn Biology, R1706-7), anti-ADSS (1:500, ABclonal, A3720), anti-IMPDH2 (1:2000, Proteintech, 12948-1-AP), anti-ADSL (1:1000, Abcam, ab154182), anti-HPRT (1:500, Abcam, ab10479), anti-APRT (1:400, ABclonal, A5456), and anti-β-actin (1:5000, Sigma, ab8227) as an internal control. The primary antibodies were revealed using the appropriate secondary antibody conjugated to peroxidase and enhanced chemiluminescence (Thermo Fisher Scientific, Waltham, MA, USA).

### RNA arrays

GSC2 cells were digested by Accutase (Gibco, Carlsbad, CA, USA), and total RNA was isolated from the lysed cells with TRIzol reagent (Invitrogen). RNA integrity was determined by capillary electrophoresis using the RNA 6000 Nano Lab-on-a-Chip kit and Bioanalyzer 2100 (Agilent Technologies, Santa Clara, CA, USA). Total RNA was amplified and labeled using a CapitalBio cRNA amplification and labeling kit (CapitalBio). Labeled cDNA was purified with a PCR NucleoSpin Extract II kit (MN) and hybridized to the CapitalBio Technology Human LncRNA Array v4.0, 4x180K array (CapitalBio Technology Corporation).

## Results

### Upregulation of purine and pyrimidine metabolism in response to acidosis stress

To understand how acidosis alters the metabolic phenotype of GSCs, unbiased metabolomic and target metabolomic analysis were performed with GSC2 under acidic and normal growth conditions. We previously determined GSCs grew optimally at an acidic pH of 6.8 (ref. ^29^). Therefore, in the present study, pH 6.8 and 7.4 were established as the acidic and normal conditions. OPLS-DA and variable importance in the projection analysis revealed a set of metabolites that discriminated between GSC2 cultured at pH 6.8 and pH 7.4. In total, 74 metabolites that were statistically significantly different in the two pH conditions were identified based on a threshold of VIP values >2, an independent *t*-test (*p* < 0.05), and an intensity fold-change of at least 1.2 (up or down; Fig. S1).

**Fig. 1:**
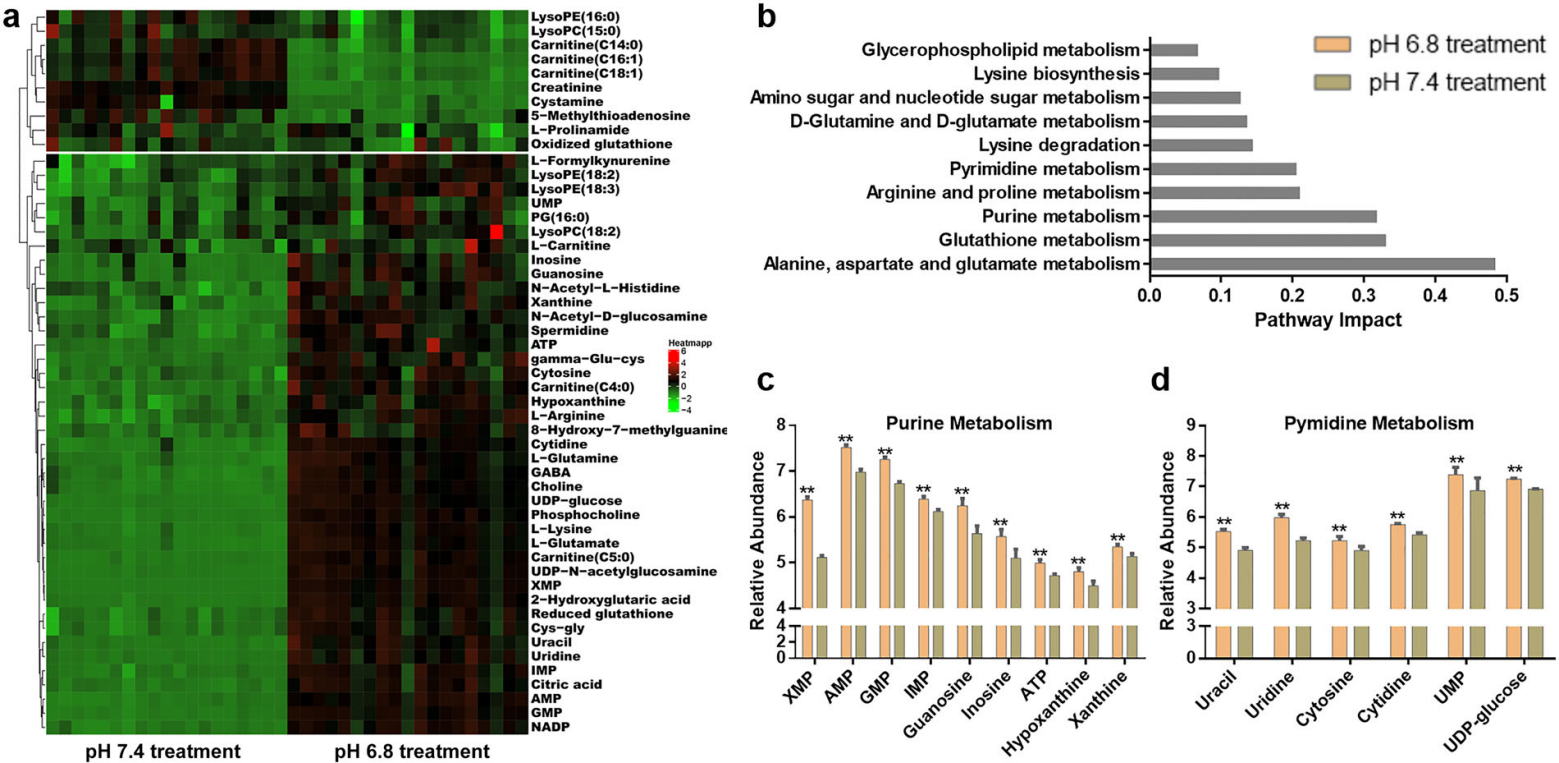
Upregulation of purine and pyrimidine metabolism in response to acidosis stress. **a** Metabolomic comparisons of GSC2 cultured at pH 6.8 and pH 7.4. **b** Pathway analysis of significantly changed (*p* < 0.05, two-tailed Student’s *t*-test) metabolites at pH 6.8. **c** Relative abundances of the significantly changed purine metabolites in GSC2 as determined by LC–MS. (**p < 0.01). **d** Relative abundances of the significantly changed pyrimidine metabolites in GSC2 as determined by LC–MS. (**p < 0.01).

Another independent set of cell samples was used in the subsequent target metabolomics analysis and yielded 51 differential metabolites that were further validated (Figs. 1a and S2 and Table S3). Of these candidates, 41 metabolites exhibited increasing abundance and 10 metabolites exhibited decreasing abundance in GSC2 cultured at pH 6.8 compared with those cultured at pH 7.4. Further, metabolic pathway analysis of the 51 differential metabolites suggested profound alterations of alanine, aspartate, and glutamate metabolism; glutathione metabolism; purine metabolism; arginine and proline metabolism, and pyrimidine metabolism, among others. The significantly changed pathways (pathway impact >0.1) are shown in Fig. 1b. Accompanying a high ratio of GSH/GSSH, intermediates in glutathione metabolism (gamma-glu-cys, cys-gly, spermidine, and L-glutamate) accumulated in GSC2 under acidic conditions (Fig. S3). Nucleotides, nucleosides, and nucleobases, including nine purine metabolites and six pyrimidine metabolites, were also dramatically increased in GSC2 cultured under acidic conditions (Fig. 1c, d).

### Increased energy metabolism in an acidic microenvironment

To determine the effect of low pH on energy metabolism-related pathways, we performed metabolic flux analysis using ^13^C_6_-glucose as a tracer to examine the TCA cycle of GSC2 cultured at pH 6.8 and pH 7.4. The abundances of the intermediates from energy metabolism-related pathways were significantly increased, including citrate/isocitrate, α-ketoglutarate, and succinate (Fig. S7).

**Fig. 2:**
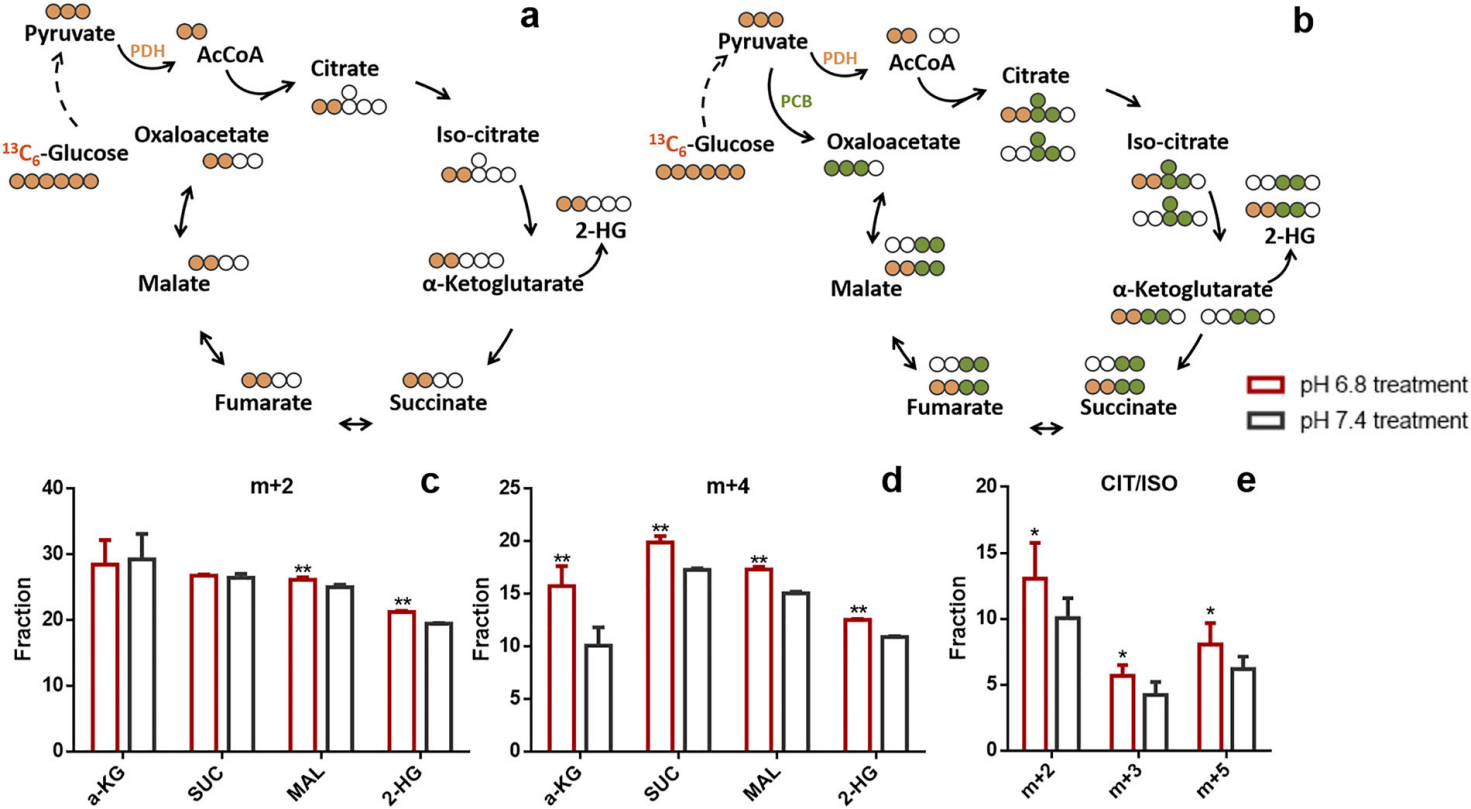
Increased energy metabolism in an acidic microenvironment. **a**, **b** Schematic of ^13^C_6_-labeled glucose as it progresses through the TCA cycle. Only a subset of possible labeled metabolites is shown. White circle: ^12^C; orange circle, green circle: ^13^C derived from PDH- and PCB-mediated Krebs cycle reactions, respectively. **c**–**e** Fraction (%) of metabolites in the TCA cycle in GSC2 cultured at pH 6.8 and pH 7.4.

In anaerobic glycolysis, ^13^C_6_-glucose generates pyruvate (m + 3) and further forms lactate (m + 2). Pyruvate (m + 3) can generate acetyl-CoA (m + 2) through pyruvate dehydrogenase (PDH), which combines with oxaloacetate (OAA) to generate ^13^C_2_-labeled intermediates of TCA cycle (Fig. 2a)^38^. Conversely, pyruvate carboxylase (PCB) carboxylates pyruvate (m + 3) with unlabeled CO_2_, forming OAA (m + 3), which combines with either unlabeled or ^13^C_2_-labeled acetyl-CoA to generate citrate (m + 3) or citrate (m + 5), and other labeled TCA cycle intermediates (Fig. 2b)^39^. The possible labeled metabolites generated through PDH and PCB are shown in Fig. 2a, b, respectively. Strikingly, elevated TCA cycle flux was observed in pH 6.8 treated GSC2, consistent with ^13^C_6_-glucose labeling of CIT/ISO (m + 2, m + 3, m + 5), α-KG (m + 2, m + 4), SUC (m + 2, m + 4), and MAL (m + 2, m + 4), under each condition (Fig. 2c–e). In addition, the fraction of 2-HG (m + 2, m + 4) also increased in pH 6.8 treated GSC2 (Fig. 2c, d).

### The effect of an acidic microenvironment on de novo nucleotide biosynthesis based on metabolic flux analysis

**Fig. 3:**
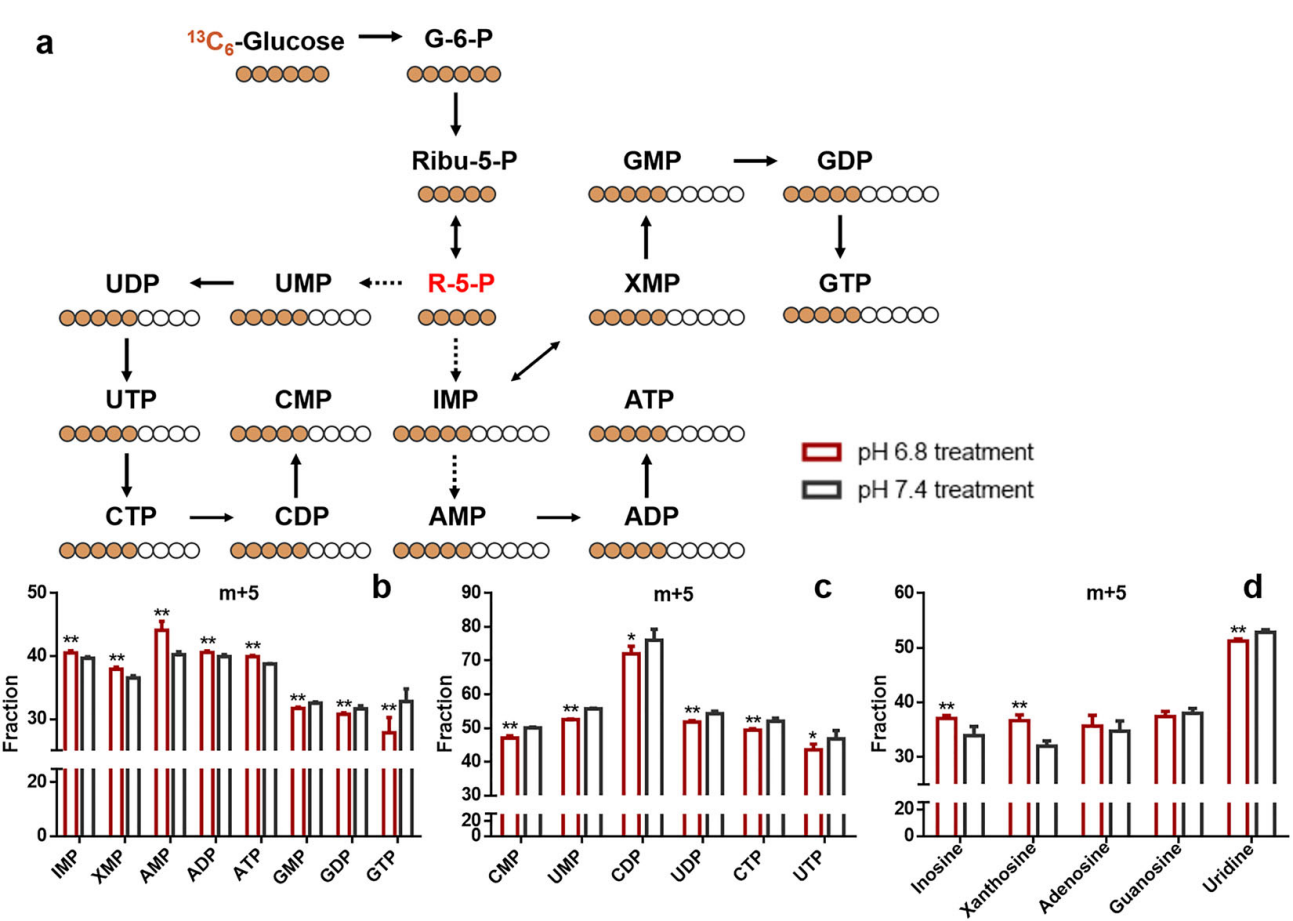
GSCs with pH 6.8 treatment have high rates of de novo purine nucleotide biosynthesis utilizing glucose-derived carbon. **a** A schematic illustrating the metabolic assimilation of glucose-carbon to nucleotide biosynthesis. **b**–**d** Fraction (%) of purine and pyrimidine metabolites in GSC2 cultured at pH 6.8 and pH 7.4.

To determine the effect of an acidic microenvironment on de novo nucleotide biosynthesis flux, we performed metabolic flux analysis using ^13^C_6_-glucose as a tracer to examine the metabolism of glucose-derived carbon atoms in GSC2 cultured at pH 6.8 and pH 7.4. Glucose-carbon can be integrated into nucleotides after it has been converted to R5P through PPP (Fig. 3a). The isotopologue distribution of IMP, XMP, AMP, GMP, CMP, UMP, and their derivatives was determined by LC–MS. Our results show that GSC2 cultured at pH 6.8 incorporated label into purine nucleotides more efficiently than GSC2 cultured at pH 7.4, except for GMP; whereas, labels were incorporated more efficiently into pyrimidines by GSC2 cultured at pH 7.4 than by GSC2 cultured at pH 6.8. (Fig. 3b–d). The high ^13^C-labeled (m + 5) fractions of IMP, XMP, AMP, and their derivatives including ADP, ATP, inosine, and xanthosine, indicate that GSC2 preferentially utilize R5P to support de novo purine nucleotide biosynthesis under acidosis; in contrast, pyrimidine synthesis flux was not increased under acidic conditions.

**Fig. 4:**
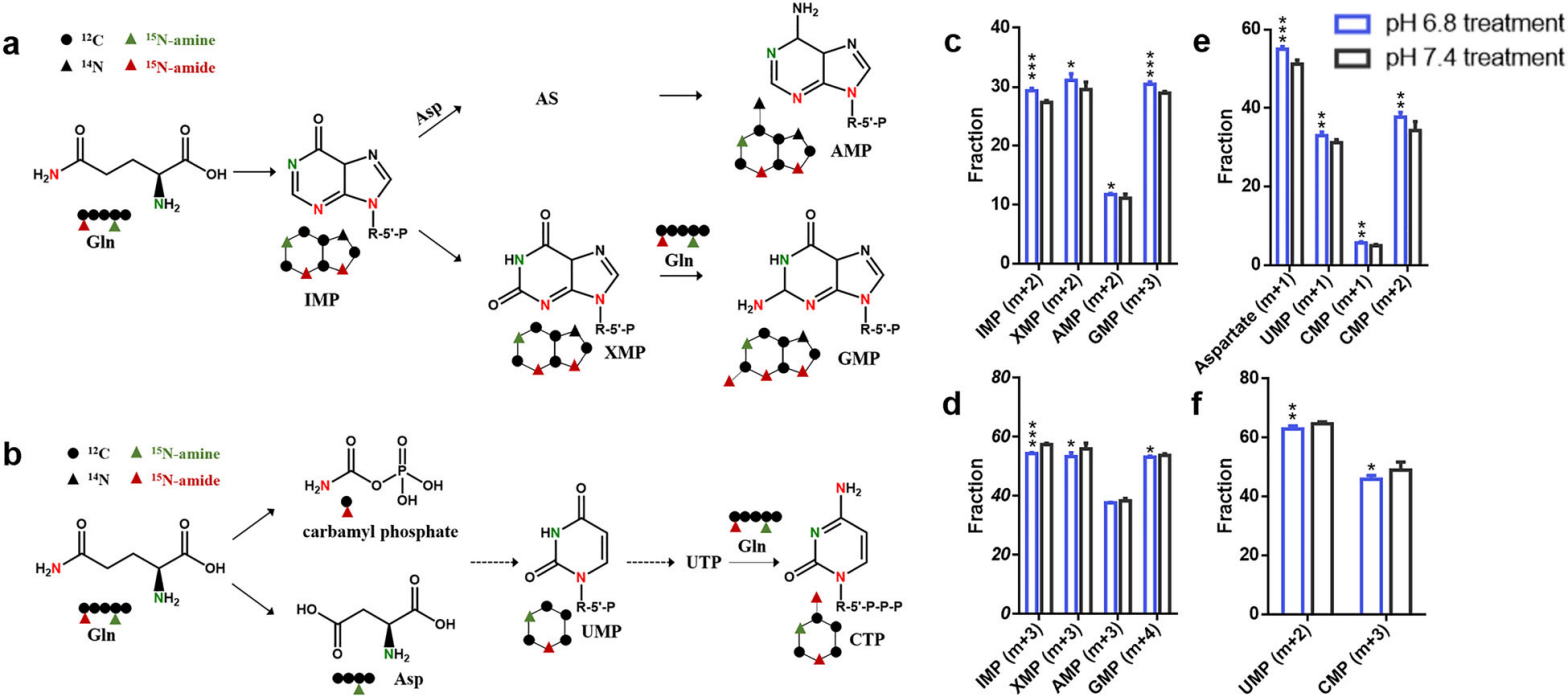
The utilization of glutamine-nitrogen in nucleotide biosynthesis. Schematic illustrating the metabolic assimilation of **a** glutamine-nitrogen to purine nucleotide biosynthesis and **b** glutamine-nitrogen to pyrimidine nucleotide biosynthesis. **c**–**f** Fraction (%) of nucleotides and aspartate in GSC2 cultured at pH 6.8 and pH 7.4.

Next, we investigated the effect of low pH on nucleotide biosynthesis rates by measuring the ^15^N from ^15^N_2_-glutamine incorporated into purine and pyrimidine nucleotides. The labeled amide of glutamine incorporates into purine nucleotides forming IMP (m + 2), XMP (m + 2), AMP (m + 2), and GMP (m + 3). In contrast, the labeled amine of glutamine can be assimilated into purine nucleotides with (m + 1) labeling form (Fig. 4a). For pyrimidine nucleotides^40^, ^15^N-amide of glutamine labeled UMP and CMP with one ^15^N atom and two ^15^N atoms, respectively. ^15^N-amine of glutamine labeled aspartate with one ^15^N atom. Subsequently, the labeled aspartate can be integrated into pyrimidine nucleotides generating UMP (m + 1) and CMP (m + 1) (Fig. 4b). In this experiment, we used ^15^N_2_-glutamine as a tracer and discriminate between ^15^N-amine and ^15^N-amide. The GSC2 cells grown under acidic conditions had higher fractions of the (m + 2) form of IMP, XMP, and AMP and the (m + 3) form of GMP (Fig. 4c). However, the labeled fractions of IMP (m + 3), XMP (m + 3), AMP (m + 3), and GMP (m + 4) were reduced under acidic conditions (Fig. 4d). For pyrimidine nucleotides, the fraction of the (m + 1) form of aspartate, UMP, and CMP, and the (m + 2) form of CMP were significantly increased in low-pH-treated GSC2 (Fig. 4e). In contrast, the fractions of UMP (m + 2) and CMP (m + 3) were reduced in pH 6.8 treated GSC2 (Fig. 4f). The labeled forms and fractions of purine and pyrimidine nucleotides might suggest an enrichment of amide-nitrogen, rather than amine-nitrogen, from glutamine in GSC2 under conditions of low pH.

### Acidosis stress enhances the expression of glucose-6-phosphate dehydrogenase

**Fig. 5:**
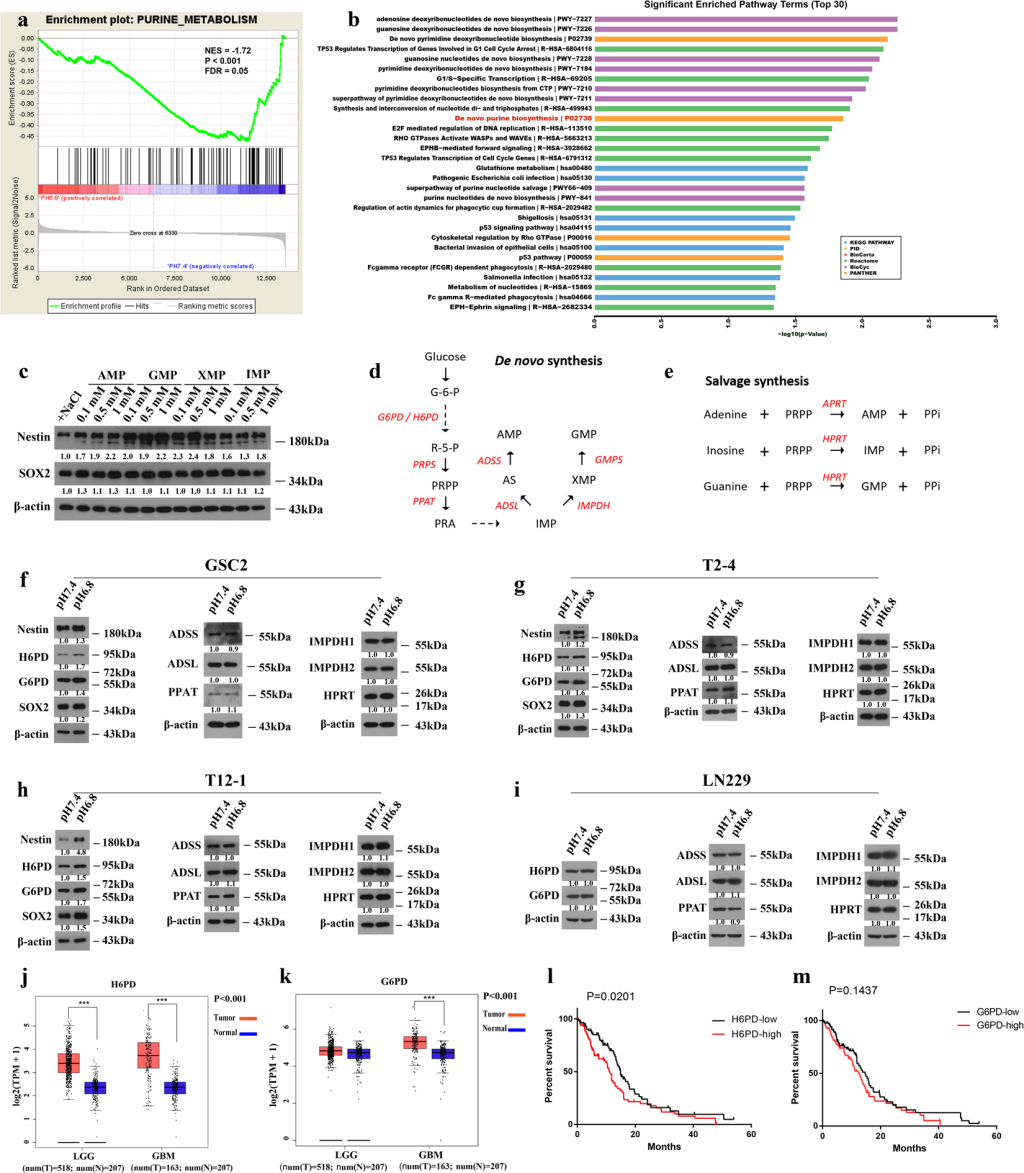
Acidosis enhances the expression of glucose-6-phosphate dehydrogenase to provide ribose-5-phosphate for nucleotide biosynthesis. **a** Gene Set Enrichment Analysis reveals the affected purine metabolism in low-pH-treated GSC2. **b** Pathway enrichment analysis in response to acidosis (top 30). **c** Expression of stemness markers in GSC2 exposed to purine nucleotide metabolites for 48 h. **d** Scheme representing the main enzymes and metabolites in the pentose phosphate pathway (PPP) and de novo purine nucleotide synthesis. **e** Schematic representing the main enzymes in the PPP and purine nucleotide salvage synthesis. **f**–**i** Western blot analysis of the expression of the above main enzymes in GSC2, T2-4, T12-1, and LN229 under pH 6.8 or pH 7.4 treatment conditions. **j**, **k** The expression levels of H6PD and G6PD in low-grade glioma (LGG) and glioblastoma (GBM) samples using the Web-based tool GEPIA. (***p < 0.001). **l**, **m** Analysis of survival based on H6PD and G6PD expression in the glioblastoma patients by The Cancer Genome Atlas (TCGA) datasets. Log-rank test. (H6PD^low^ = 84; H6PD^high^ = 84; G6PD^low^ = 84; G6PD^high^ = 83).

We analyzed gene expression data in GSC2 under different pH conditions. Consistent with the results from our metabolomic analysis, Gene Set Enrichment Analysis (GSEA) (ref. ^41^) indicated that the purine metabolic pathway was significantly affected in pH 6.8 treated GSC2 (Fig. 5a). Pathway Enrichment Analysis also demonstrated that purine metabolic pathway was significantly enriched under acidic conditions (Fig. 5b).

To further explore the critical role of purine nucleotides in GSCs, we examined the expression of stemness markers in GSC2 treated with purine nucleotides. Western blot analysis showed that the addition of AMP, GMP, XMP, and IMP to the culture medium of GSC2 promoted the expression of stemness markers (Fig. 5c). Although the protein expression levels of key enzymes involved in de novo purine synthesis and salvage synthesis were not significantly upregulated under acidic conditions (Fig. 5d, e), the protein expression levels of H6PD and G6PD in the PPP were increased in GSC2 (Fig. 5f), and the expression level of stemness markers in acidic-treated GSC2 was increased, which was consistent with our previous study^29^. In addition, we added two other GSC lines (T2-4 and T12-1). The results of T2-4 and T12-1 were consistent with GSC2 (Fig. 5g, h). However, the same result was not found with LN229 differentiated glioma cells (Fig. 5i). Collectively, these results indicate that, under acidic conditions, G6PD and H6PD may play important roles in metabolic remodeling of GSCs but not differentiated glioma cells.

The expression levels of these enzymes were compared between glioma patients group and controls using the web-based tool GEPIA. The gene expression levels of H6PD, G6PD, ADSL, APRT, GMPS, PRPS1, and IMPDH1 in human glioma patients were significantly higher than those in the control group (Figs. 5j, k and S8). To determine the clinical relevance of purine biosynthesis, we analyzed the relationship between the expression of purine synthesis enzymes and patient outcomes. The results showed that high expression of H6PD, ADSSL1, ADSS, and IMPDH1 was associated with worse prognosis for glioblastoma patients (Figs. 5l, m, S9, and S10).

## Discussion

Our previous findings showing that ATP production and mitochondrial activity were increased in GSCs in an acidic microenvironment, suggested a high level of OXPHOS (ref. ^29^). Therefore, in the present study, we focused our analysis on the mitochondrial TCA cycle, which is the hub of energy metabolism. The TCA cycle can generate reducing equivalents, including NADH and FADH2, which are transferred to the mitochondrial electron transport chain to promote OXPHOS for producing energy^42^. Our finding that TCA cycle flux was increased, lends further support for the importance of mitochondrial metabolism in GSCs under acidic conditions. Although the previous observations of Warburg suggested the existence of a glycolytic phenotype and a permanent impairment of mitochondrial OXPHOS in cancer cells, recent studies have provided a greater understanding that energy metabolism in some cancers extends beyond the Warburg effect and exists an intact mitochondrial OXPHOS (refs. ^43–45^). In the study conducted by Wheaton et al.^46^, the importance of mitochondria for cancer cell survival, growth, and proliferation was revealed as was the potential of using mitochondria as therapy targets.

Using untargeted and targeted metabolomics approaches, we found that the metabolism levels of purine, pyrimidine, and glutathione were elevated in GSCs in an acidic microenvironment. In nucleotide metabolism, including purine metabolism and pyrimidine metabolism, the levels of nucleobases, nucleosides, and nucleotides were all higher under acidic conditions than they were under normal conditions. As nucleobases and nucleosides are the products of nucleotide degradation, their elevated levels in low-pH-treated GSCs diminish the explanation that the high levels of IMP, XMP, AMP, GMP, and UMP observed was caused by the blockage in nucleotide degradation^18^. This finding suggests that nucleotides, which are required for DNA replication and RNA production, may play critical roles in the cellular response to acidosis. A recent study showed that de novo purine synthesis promoted cell resistance to radiation in GBM. It is difficult to design personalized targeted therapies because of intratumoral genomic heterogeneity; however, the metabolic changes tend to be the same, for example, purine metabolism, suggesting new ideas for targeted therapy (especially for cancers with genomic heterogeneity)^47^.

The results from our metabolic flux analysis also revealed the high utilization of carbon from glucose in purine biosynthesis under low pH conditions. The flux data suggest that acidosis drives glucose-carbon to produce R5P via the PPP to provide purine nucleotides with a sugar backbone. Unexpectedly, the results showed that acidic conditions did not promote the increased incorporation of glucose-carbon in pyrimidine nucleotides This result underscores the upregulation of de novo purine nucleotide biosynthesis in low-pH-treated GSCs. In addition, the results of the labeled forms and fractions of nucleotides following glutamine-nitrogen labeling, probably suggest that acidosis promotes the assimilation of amide-nitrogen, not amine-nitrogen, to nucleotide biosynthesis. However, to confirm this idea, additional metabolic flux analysis using both ^15^N-amide-glutamine and ^15^N-amine-glutamine as tracers is needed.

The GSEA of gene expression microarray datasets also revealed significant enrichment of gene sets related to purine metabolism in pH 6.8 treated GSCs, which supports our findings at the metabolic level. Western blot results confirmed that purine nucleotides, including IMP, XMP, GMP, and AMP, contribute to the maintenance of GSCs stemness in an acidic microenvironment. Similarly, global metabolomic and genomic analysis by Wang et al. demonstrated the dependency of glioma tumor-initiating cells on de novo purine synthesis, as well as on increased levels of purine synthesis enzymes^18^. However, in our study, the protein expression levels of enzymes involved in de novo purine nucleotide synthesis and salvage purine synthesis were not significantly upregulated under acidic conditions. We speculated that this outcome might be the result of changes in the expression of other enzymes upstream of purine nucleotide synthesis.

As a branch at the first committed step of glycolysis, the PPP plays a pivotal role in maintaining tumor cell growth by providing cells with ribose-5-phosphate for de novo nucleotide biosynthesis and NADPH for regulating the level of GSH (ref. ^48^). Our results revealed an interaction between an acidic microenvironment and upregulated GSH metabolism in GSCs. Together, the upregulation of GSH and purine nucleotides suggests that metabolic reprogramming in GSCs may be initiated by the upregulation of PPP under acidic conditions. Consistent with this idea, the expression level of glucose-6 phosphate dehydrogenase, a key enzyme in the PPP, was increased in the GSCs cultured at pH 6.8. Zhang et al.^49^ found that G6PD mediated cellular antioxidant defense and upregulation of nucleotide generation led to tumorigenic transformation in mouse and human cells. Many studies have reported that the aberrant activation of G6PD or H6PD is related to tumorigenesis and malignancy in rapidly growing cancer cells^50–53^. Studies^54,55^ have also found that the downregulation of H6PD can affect the proliferation and migration of colon and breast cancer cells.

Drug, radiation, and chemotherapy resistance correlates with high levels of GSH, and nucleotides, and activated PPP in multiple cancer cells^56–59^. These findings suggest that it might be possible to directly inhibit the upstream PPP to break redox homeostasis and block nucleotide synthesis, thus selectively eradicating cancer cells. An inhibitor of the second enzyme of the PPP, which is a combination of 2-deoxy-D-glucose, a glucose analog, and 6-aminonicotinamide, has been shown to enhance radio-sensitivity in human gliomas^60^. These findings suggest that GSCs may be sensitive to inhibition of G6PD and H6PD. By analyzing relevant genes in the TCGA and Chinese Glioma Genome Atlas, we found that the expression of G6PD and H6PD, the two genes encoding glucose-6 phosphate dehydrogenase, was increased in glioma patients, particularly H6PD. In addition, we found that H6PD, but not G6PD, was associated with a poor survival rate for glioblastoma patients. However, the relationship between G6PD, H6PD, and purine metabolism in GSCs under acidic microenvironment requires further study.

Moreover, emerging studies have revealed that metabolic alterations in glioblastoma may contribute to immunosuppression, thereby representing a series of metabolic immune checkpoints^61^. Aberrant tryptophan metabolism has been identified as an important metabolic node and immune checkpoint in glioblastoma, and several studies have discovered that targeting this pathway’s rate-limiting enzyme indoleamine 2,3-dioxygenase 1 can enhance therapeutic effect by mitigating immunosuppression^62–64^. The acidic microenvironment plays a contributory role in immune regulation and cancer progression by affecting various components of tumor immune surveillance^65^. Exposing to the low pH environment leads to the dysfunction of antitumor effectors such as NK and T cells and the activation of immunosuppressive components such as regulatory T cells and myeloid cells^65–69^. Therefore, we believe that reversing of the metabolic remodeling of GSCs driven by acidosis, especially by targeting the rate-limiting enzyme G6PD or H6PD, may potentially eliminate the immunosuppressive effect.

**Fig. 6:**
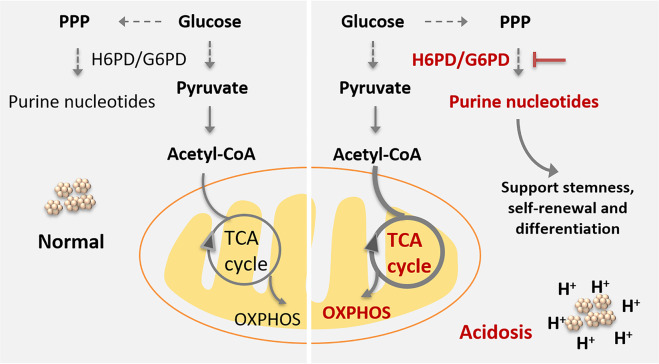
Model of metabolic rewiring in GSCs in response to acidosis stress. In response to acidosis stress, GSCs exhibited increased de novo purine nucleotide biosynthesis pathway, which is under the regulation of glucose-6-phosphate dehydrogenase. The overexpression G6PD/H6PD, supports the metabolic dependency of GSCs on nucleotides by enhancing the pentose phosphate pathway (PPP). In parallel, TCA cycle flux was also increased, lends support for the importance of mitochondrial metabolism in GSCs under acidic conditions. This reprogramming of GSCs metabolism driven by acidosis indicates that targeting G6PD/H6PD may serve as a promising therapeutic target for improved glioblastoma therapeutics.

Taken together, our work describes a metabolic rewiring model of GSCs in an acidic microenvironment, that is mediated primarily by the PPP, which upregulates purine nucleotide biosynthesis and increases GSH levels (Fig. 6). Although further research is needed, the present study suggests that targeting pH-sensing pathways is a promising approach to anti-GSC therapy. Key metabolic enzymes, such as H6PD or G6PD, may be potential targets for improving immune therapies and outcomes for glioma patients.

## Supplementary information

Supplementary Information

## References

[1] Hanahan D, Weinberg RA (2011). Hallmarks of cancer: the next generation. Cell.

[2] Cantor JR, Sabatini DM (2012). Cancer cell metabolism: one hallmark, many faces. Cancer Discov..

[3] Ward PS, Thompson CB (2012). Metabolic reprogramming: a cancer hallmark even warburg did not anticipate. Cancer Cell..

[4] Sun L, Suo C, Li S-t, Zhang H, Gao P (2018). Metabolic reprogramming for cancer cells and their microenvironment: beyond the Warburg effect. Biochim Biophys. Acta.

[5] Le A, Udupa S, Zhang C (2019). The metabolic interplay between cancer and other diseases. Trends Cancer.

[6] Luo J, Solimini NL, Elledge SJ (2009). Principles of cancer therapy: oncogene and non-oncogene addiction. Cell.

[7] Tennant DA, Duran RV, Gottlieb E (2010). Targeting metabolic transformation for cancer therapy. Nat. Rev. Cancer.

[8] Vander Heiden MG (2011). Targeting cancer metabolism: a therapeutic window opens. Nat. Rev. Drug Discov..

[9] El-Habr EA (2017). A driver role for GABA metabolism in controlling stem and proliferative cell state through GHB production in glioma. Acta Neuropathol..

[10] Stupp R (2009). Effects of radiotherapy with concomitant and adjuvant temozolomide versus radiotherapy alone on survival in glioblastoma in a randomised phase III study: 5-year analysis of the EORTC-NCIC trial. Lancet Oncol..

[11] Alifieris C, Trafalis DT (2015). Glioblastoma multiforme: pathogenesis and treatment. Pharm. Ther..

[12] Singh SK (2004). Identification of human brain tumour initiating cells. Nature.

[13] Bao S (2006). Glioma stem cells promote radioresistance by preferential activation of the DNA damage response. Nature.

[14] Thomas TM, Yu JS (2017). Metabolic regulation of glioma stem-like cells in the tumor micro-environment. Cancer Lett..

[15] Chen J (2012). A restricted cell population propagates glioblastoma growth after chemotherapy. Nature.

[16] Wang R (2010). Glioblastoma stem-like cells give rise to tumour endothelium. Nature.

[17] Vlashi E (2011). Metabolic state of glioma stem cells and nontumorigenic cells. Proc. Natl Acad. Sci. USA.

[18] Wang X (2017). Purine synthesis promotes maintenance of brain tumor initiating cells in glioma. Nat. Neurosci..

[19] Zhang R (2017). LC-MS-based metabolomics reveals metabolic signatures related to glioma stem-like cell self-renewal and differentiation. RSC Adv..

[20] Li Z (2009). Hypoxia-inducible factors regulate tumorigenic capacity of glioma stem cells. Cancer Cell.

[21] Calabrese C (2007). A perivascular niche for brain tumor stem cells. Cancer Cell.

[22] Schonberg DL, Bao S, Rich JN (2013). Genomics informs glioblastoma biology. Nat. Genet..

[23] Hjelmeland AB (2011). Acidic stress promotes a glioma stem cell phenotype. Cell Death Differ..

[24] Damaghi M, Wojtkowiak JW, Gillies RJ (2013). pH sensing and regulation in cancer. Front Physiol..

[25] Corbet C, Feron O (2017). Tumour acidosis: from the passenger to the driver’s seat. Nat. Rev. Cancer.

[26] Haley EM, Tilson SG, Triantafillu UL, Magrath JW, Kim Y (2017). Acidic pH with coordinated reduction of basic fibroblast growth factor maintains the glioblastoma stem cell-like phenotype in vitro. J. Biosci. Bioeng..

[27] Zhang D (2019). Metabolic regulation of gene expression by histone lactylation. Nature.

[28] Khacho M (2014). Acidosis overrides oxygen deprivation to maintain mitochondrial function and cell survival. Nat. Commun..

[29] Hu PS (2019). Acidosis enhances the self-renewal and mitochondrial respiration of stem cell-like glioma cells through CYP24A1-mediated reduction of vitamin D. Cell Death Dis..

[30] Corbet C (2016). Acidosis drives the reprogramming of fatty acid metabolism in cancer cells through changes in mitochondrial and histone acetylation. Cell Metab..

[31] Abrego J (2017). GOT1-mediated anaplerotic glutamine metabolism regulates chronic acidosis stress in pancreatic cancer cells. Cancer Lett..

[32] Hu PS (2017). NSPc1 promotes cancer stem cell self-renewal by repressing the synthesis of all-trans retinoic acid via targeting RDH16 in malignant glioma. Oncogene.

[33] Xu XY (2019). Systematic optimization and evaluation of sample pretreatment methods for LC-MS-based metabolomics analysis of adherent mammalian cancer cells. Anal. Methods.

[34] Smith CA, Want EJ, O’Maille G, Abagyan R, Siuzdak G (2006). XCMS: processing mass spectrometry data for metabolite profiling using Nonlinear peak alignment, matching, and identification. Anal. Chem..

[35] Xia JG, Sinelnikov IV, Han B, Wishart DS (2015). MetaboAnalyst 3.0-making metabolomics more meaningful. Nucleic Acids Res..

[36] Wishart DS (2013). HMDB 3.0—the human metabolome database in 2013. Nucleic Acids Res..

[37] Smith CA (2005). METLIN: a metabolite mass spectral database. Ther. Drug Monit..

[38] Bruntz RC, Lane AN, Higashi RM, Fan TW (2017). Exploring cancer metabolism using stable isotope-resolved metabolomics (SIRM). J. Biol. Chem..

[39] Buescher JM (2015). A roadmap for interpreting (13)C metabolite labeling patterns from cells. Curr. Opin. Biotechnol..

[40] Wang Y (2019). Coordinative metabolism of glutamine carbon and nitrogen in proliferating cancer cells under hypoxia. Nat. Commun..

[41] Subramanian A (2005). Gene set enrichment analysis: a knowledge-based approach for interpreting genome-wide expression profiles. Proc. Natl Acad. Sci. USA.

[42] Hatefi Y (1985). The mitochondrial electron-transport and oxidative-phosphorylation system. Annu Rev. Biochem..

[43] Cairns RA, Harris IS, Mak TW (2011). Regulation of cancer cell metabolism. Nat. Rev. Cancer.

[44] Zheng J (2012). Energy metabolism of cancer: glycolysis versus oxidative phosphorylation (Review). Oncol. Lett..

[45] Frezza C, Gottlieb E (2009). Mitochondria in cancer: not just innocent bystanders. Semin Cancer Biol..

[46] Wheaton WW (2014). Metformin inhibits mitochondrial complex I of cancer cells to reduce tumorigenesis. Elife.

[47] Zhou W (2020). Purine metabolism regulates DNA repair and therapy resistance in glioblastoma. Nat. Commun..

[48] Lane AN, Fan TW (2015). Regulation of mammalian nucleotide metabolism and biosynthesis. Nucleic Acids Res..

[49] Zhang Y (2021). Upregulation of antioxidant capacity and nucleotide precursor availability suffices for oncogenic transformation. Cell Metab..

[50] Zhang HS (2019). Nrf2 promotes breast cancer cell migration via up-regulation of G6PD/HIF-1alpha/Notch1 axis. J. Cell Mol. Med..

[51] Wu SR (2018). Transcription factor YY1 promotes cell proliferation by directly activating the pentose phosphate pathway. Cancer Res..

[52] Wu YH (2018). Glucose-6-phosphate dehydrogenase is indispensable in embryonic development by modulation of epithelial-mesenchymal transition via the NOX/Smad3/miR-200b axis. Cell Death Dis..

[53] Chen XY (2018). Modulation of G6PD affects bladder cancer via ROS accumulation and the AKT pathway in vitro. Int. J. Oncol..

[54] Tsachaki M, Mladenovic N, Stambergova H, Birk J, Odermatt A (2018). Hexose-6-phosphate dehydrogenase controls cancer cell proliferation and migration through pleiotropic effects on the unfolded-protein response, calcium homeostasis, and redox balance. FASEB J..

[55] Marini C (2016). Discovery of a novel glucose metabolism in cancer: the role of endoplasmic reticulum beyond glycolysis and pentose phosphate shunt. Sci. Rep..

[56] Riganti C, Gazzano E, Polimeni M, Aldieri E, Ghigo D (2012). The pentose phosphate pathway: an antioxidant defense and a crossroad in tumor cell fate. Free Radic. Biol. Med..

[57] Obrist F (2018). Metabolic vulnerability of cisplatin-resistant cancers. EMBO J..

[58] Wang JX (2012). Overexpression of G6PD is associated with poor clinical outcome in gastric cancer. Tumor Biol..

[59] Friesen C, Kiess Y, Debatin KM (2004). A critical role of glutathione in determining apoptosis sensitivity and resistance in leukemia cells. Cell Death Differ..

[60] Manganelli G, Masullo U, Passarelli S, Filosa S (2013). Glucose-6-phosphate dehydrogenase deficiency: disadvantages and possible benefits. Cardiovasc. Hematol. Disord. Drug Targets.

[61] Kesarwani P, Kant S, Prabhu A, Chinnaiyan P (2017). The interplay between metabolic remodeling and immune regulation in glioblastoma. Neuro. Oncol..

[62] Vacchelli E (2014). Trial watch: IDO inhibitors in cancer therapy. Oncoimmunology.

[63] Wainwright DA (2014). Durable therapeutic efficacy utilizing combinatorial blockade against IDO, CTLA-4, and PD-L1 in mice with brain tumors. Clin. Cancer Res..

[64] Kesarwani P (2018). Tryptophan metabolism contributes to radiation-induced immune checkpoint reactivation in glioblastoma. Clin. Cancer Res..

[65] Huber V (2017). Cancer acidity: an ultimate frontier of tumor immune escape and a novel target of immunomodulation. Semin Cancer Biol..

[66] Calcinotto A (2012). Modulation of microenvironment acidity reverses anergy in human and murine tumor-infiltrating T lymphocytes. Cancer Res..

[67] Haas R (2015). Lactate regulates metabolic and pro-inflammatory circuits in control of T cell migration and effector functions. PLoS Biol..

[68] Facciabene A (2011). Tumour hypoxia promotes tolerance and angiogenesis via CCL28 and T-reg cells. Nature.

[69] Brand A (2016). LDHA-associated lactic acid production blunts tumor immunosurveillance by T and NK cells. Cell Metab..

