# Supplementary material for: Rewiring of purine metabolism in response to acidosis stress in glioma stem cells
Source: Cell Death Dis. 2021 Mar 15;12(3):277. doi: 10.1038/s41419-021-03543-9 (PMC7961141; doi:10.1038/s41419-021-03543-9)
Supplement: Supplementary file 1 — Supplementary Information [file 41419_2021_3543_MOESM1_ESM.docx]

**Supplementary Information**

**Rewiring of** **purine metabolism in response to** **acidosis stress in glioma stem cells**

Xiaoyu Xu^a,1^, Liping Wang^b,1^, Qingce Zang^a^, Shanshan Li^b^, Limei Li^a^, Zhixing Wang^b^, Jiuming He^a^, Boqin Qiang^b^, Wei Han^b^, Ruiping Zhang^a^, Xiaozhong Peng^*b,d^, Zeper Abliz^*a,c^

^a^State Key Laboratory of Bioactive Substance and Function of Natural Medicines, Institute of Materia Medica, Chinese Academy of Medical Sciences and Peking Union Medical College, Beijing 100050, China.

^b^State Key Laboratory of Medical Molecular Biology, Department of Molecular Biology and Biochemistry, Institute of Basic Medical Sciences, Biomedical Primate Research Center, Neuroscience Center Chinese Academy of Medical Sciences, School of Basic Medicine Peking Union Medical College, Beijing 100005, China.

^c^Centre for Bioimaging and Systems Biology, Minzu University of China, Beijing 100081, China.

^d^Institute of Medical Biology, Chinese Academy of Medical Sciences and Peking Union Medical College, Kunming 650031, China.

^1^Authors contribute to the manuscript equally.

^*^Corresponding authors.

**Checklist of Supplementary Material:**

**1) Supplementary Figure.**

**Fig.S1** Multivariate data analysis for GSC2 with pH 6.8 treatment (N=15) and pH 7.4 treatment (N=23). PCA score plots based on the LC-(+)ESI/MS (A) and LC-(-)ESI/MS (B) datasets. OPLS-DA score plots based on the LC-(+)ESI/MS (C) and LC-(-)ESI/MS (D) datasets. Plots of permutation tests of the OPLS-DA models are based on the LC-(+)ESI/MS (E) and LC-(-)ESI/MS (F) data sets. Red circle: GSC2 with pH 6.8 treatment; Blue circle: GSC2 with pH 7.4 treatment.

**Fig.S2** The typical XICs of the LC-MS/MS MRM analysis of GSC2 in positive ion mode (A) and in negative mode (B).

**Fig.S3** Relative abundance of significantly changed glutathione and related metabolites in GSC2 as determined by LC–MS (N=19, ****p* < 0.001).

**Fig.S4** The steady-state time of ^13^C enrichment of representative metabolites in GSC2 cultured with ^13^C_6_-glucose medium. (A-C) Metabolites in the glycolytic pathway. (D-E) Metabolites in the TCA cycle. (G-I) Metabolites in the PPP.

**Fig.S5** The steady-state time of ^13^C enrichment of representative metabolites in GSC2 cultured with ^13^C_6_-glucose medium. (A-C) Nucleoside monophosphate. (D-E) Nucleoside diphosphate. (G-I) Nucleoside triphosphate.

**Fig.S6** The steady-state time of ^13^C enrichment of representative metabolites in GSC2 cultured with ^13^C_6_-glucose medium. (A-C) Nucleosides. (D-E) Nucleobases. (G-I) Other metabolites related to energy metabolism.

**Fig.S7** Fold change in abundance of intermediates in the TCA cycle relative to pH 7.4 treatment as determined by LC-MS (N=6, **p* < 0.05, ***p* < 0.01).

**Fig.S8** The expression levels of ADSL,PRPS1, GMPS, IMPDH2 and APRT in both LGG and GBM samples according to the web-based tool GEPIA.

**Fig.S9** Analysis of glioblastoma patient survival based on ADSSL1 and IMPDH1 expression as indicated by TCGA datasets. Log-rank test. (ADSSL1^low^ = 83; ADSSL1^high^ =84; IMPDH1^low^ = 83; IMPDH1^high^ =84).

**Fig.S10** Analysis of glioblastoma patient survival based on H6PD, G6PD, ADSS and PRPS2 expression according to CGGA datasets. Log-rank test. (H6PD^low^ = 73; H6PD^high^ =65; G6PD^low^ = 73; G6PD^high^ =76; ADSS ^low^ = 63; ADSS^high^ =70; PRPS2^low^ = 70; PRPS2^high^ =66).

**2) Supplementary Table.**

**Table S1** Parameters of the LC-MS/MS-based targeted metabolomic analysis of GSC2 in positive ion mode.

**Table S2** The parameters of the LC-MS/MS-based targeted metabolomic analysis of GSC2 in negative ion mode.

**Table S3** Differential metabolites of GSC2 cultured under different pH conditions.

**1) Supplementary Figure.**

**
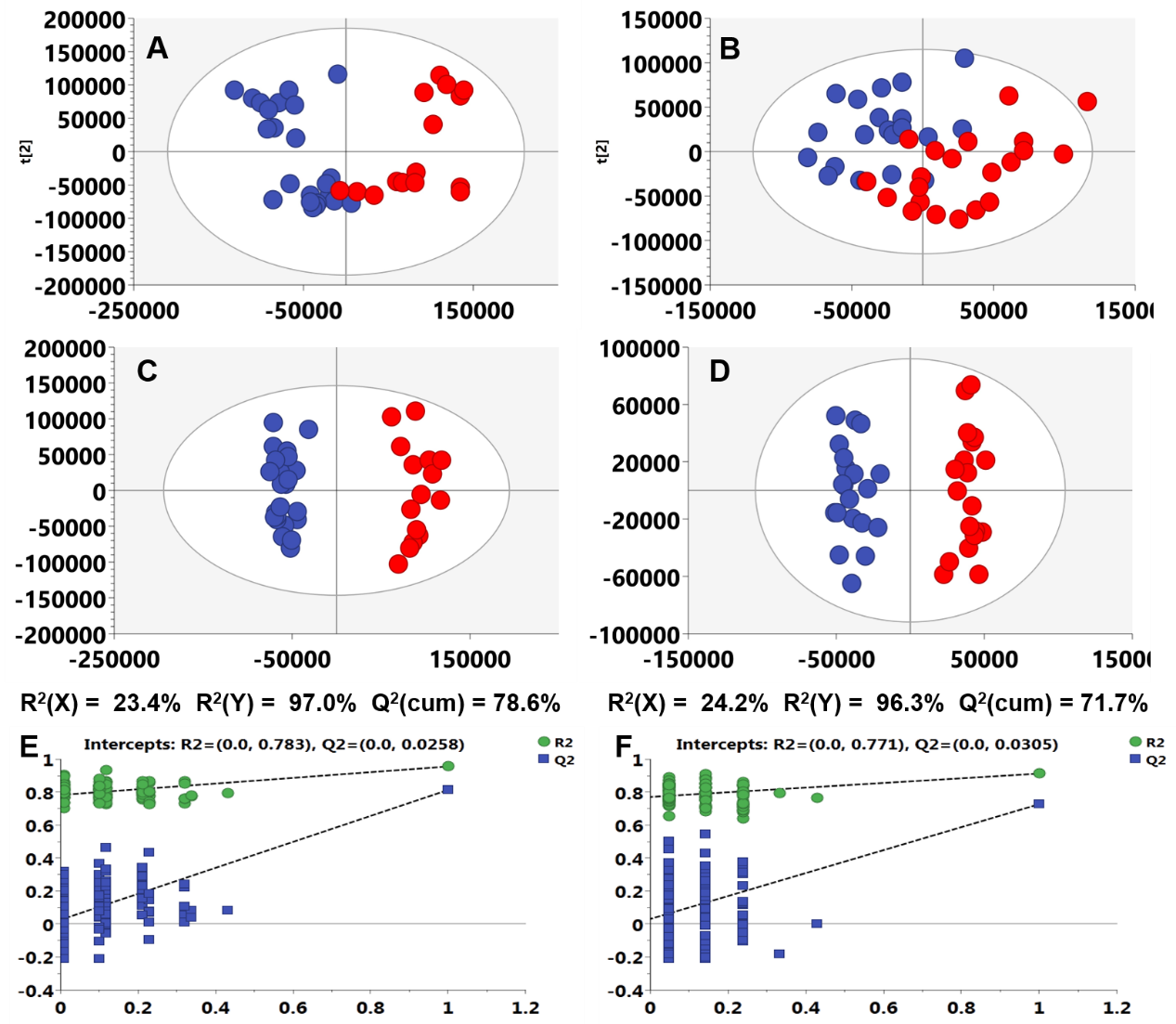
**

**Fig.S1** Multivariate data analysis for GSC2 with pH 6.8 treatment (N=15) and pH 7.4 treatment (N=23). PCA score plots based on the LC-(+)ESI/MS (A) and LC-(-)ESI/MS (B) datasets. OPLS-DA score plots based on the LC-(+)ESI/MS (C) and LC-(-)ESI/MS (D) datasets. Plots of permutation tests of the OPLS-DA models are based on the LC-(+)ESI/MS (E) and LC-(-)ESI/MS (F) data sets. Red circle: GSC2 with pH 6.8 treatment; Blue circle: GSC2 with pH 7.4 treatment.


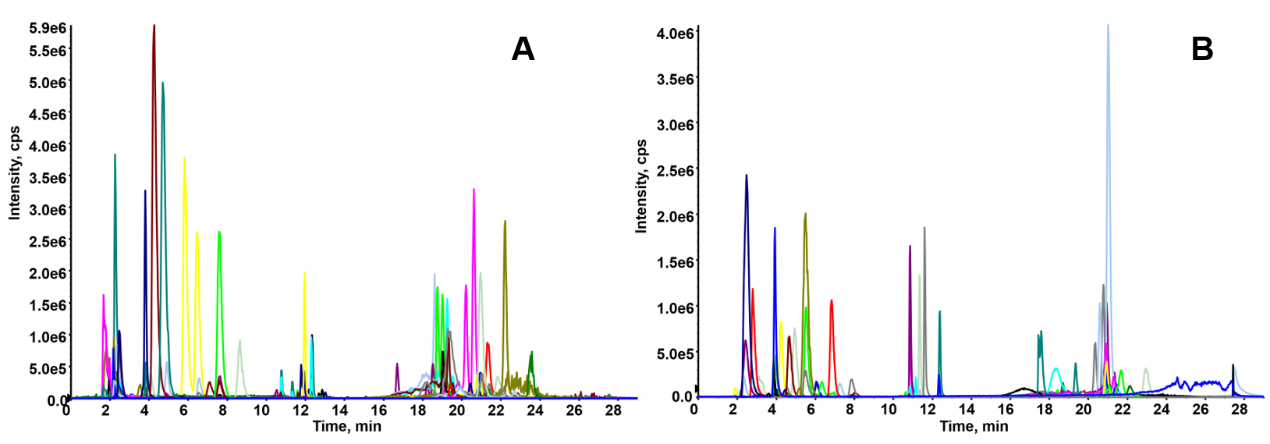


**Fig.S2** The typical XICs of the LC-MS/MS MRM analysis of GSC2 in positive ion mode (A) and in negative mode (B).

**
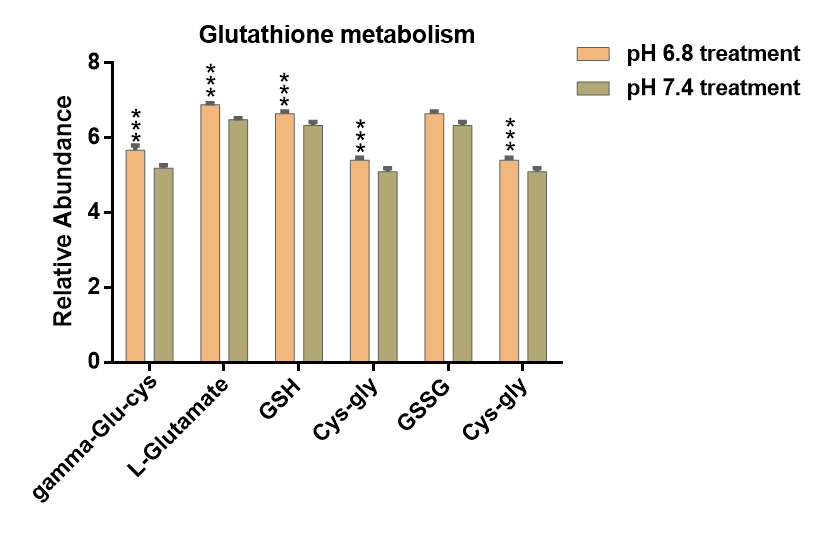
**

**Fig.S3** Relative abundance of significantly changed glutathione and related metabolites in GSC2 as determined by LC–MS (N=19, ****p* < 0.001).

**
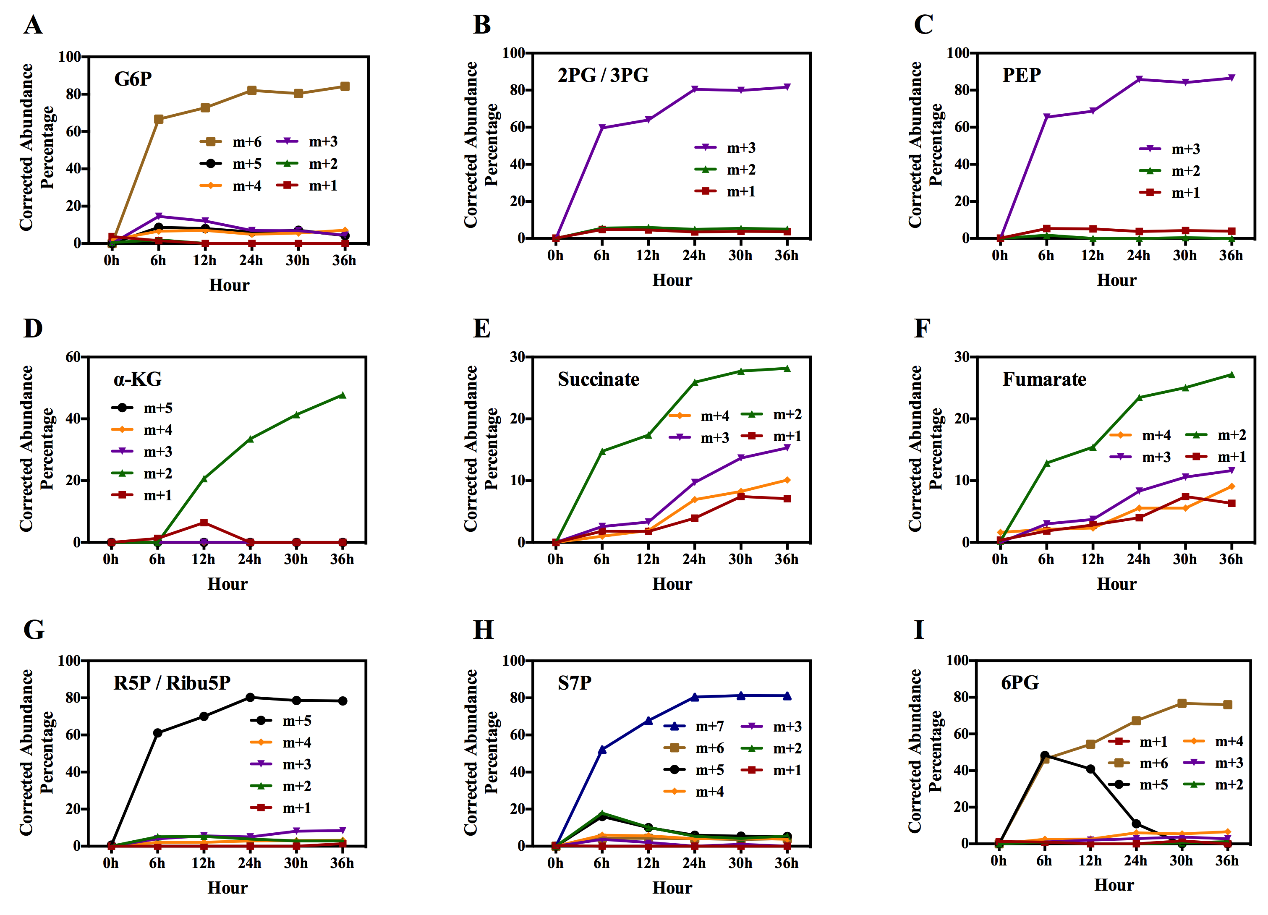
**

**Fig.S4** The steady-state time of ^13^C enrichment of representative metabolites in GSC2 cultured with ^13^C_6_-glucose medium. (A-C) Metabolites in the glycolytic pathway. (D-E) Metabolites in the TCA cycle. (G-I) Metabolites in the PPP.

**Fig.S5** The steady-state time of ^13^C enrichment of representative metabolites in GSC2 cultured with ^13^C_6_-glucose medium. (A-C) Nucleoside monophosphate. (D-E) Nucleoside diphosphate. (G-I) Nucleoside triphosphate.

**
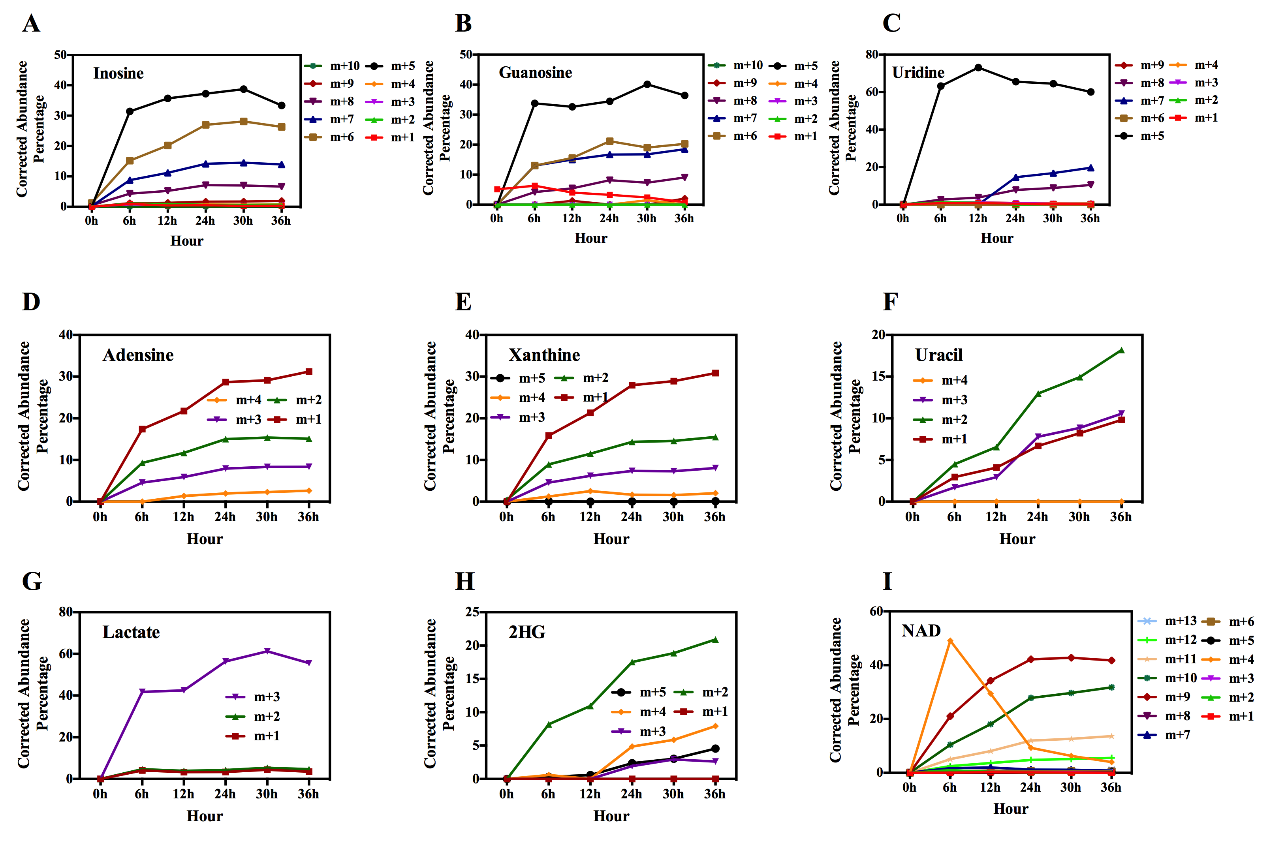
**

**Fig.S6** The steady-state time of ^13^C enrichment of representative metabolites in GSC2 cultured with ^13^C_6_-glucose medium. (A-C) Nucleosides. (D-E) Nucleobases. (G-I) Other metabolites related to energy metabolism.

**
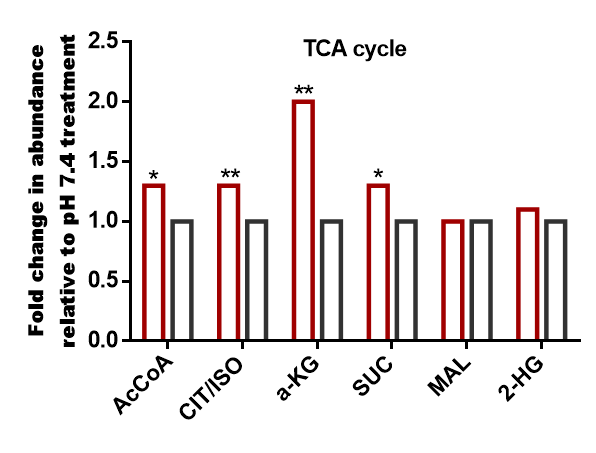
**

**Fig.S7** Fold change in abundance of intermediates in the TCA cycle relative to pH 7.4 treatment as determined by LC-MS (N=6, **p* < 0.05, ***p* < 0.01).


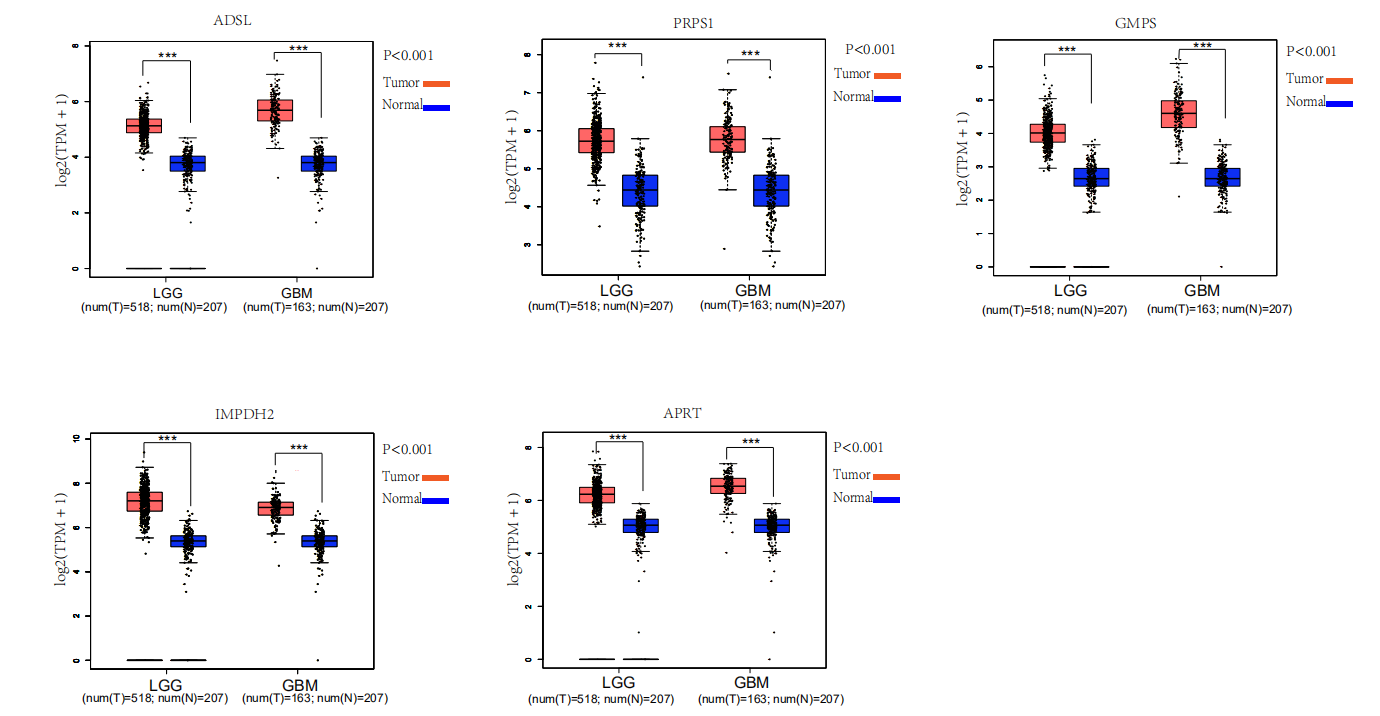


**Fig.S8** The expression levels of ADSL,PRPS1, GMPS, IMPDH2 and APRT in both LGG and GBM samples according to the web-based tool GEPIA.


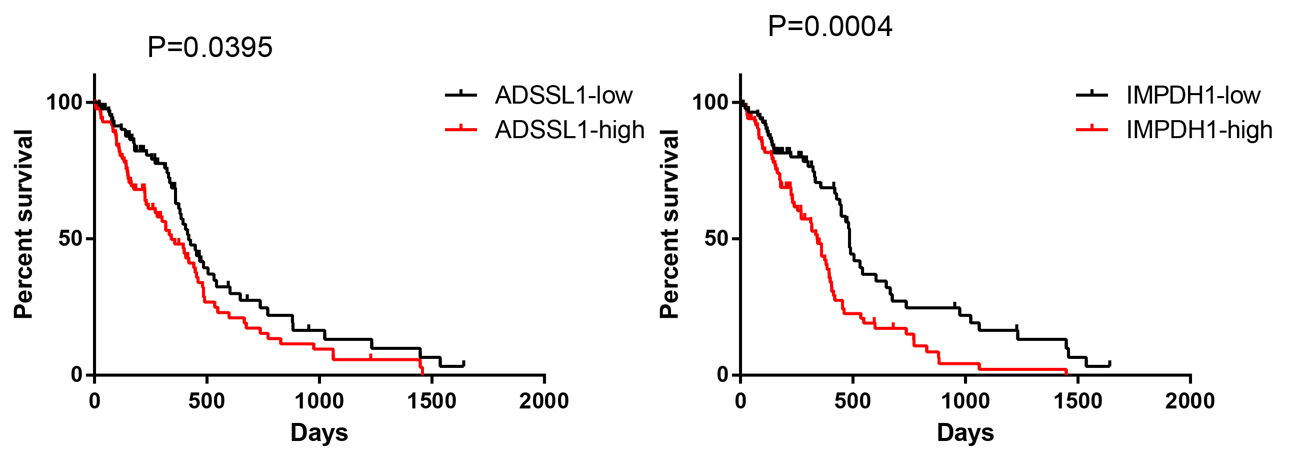


**Fig.S9** Analysis of glioblastoma patient survival based on ADSSL1 and IMPDH1 expression as indicated by TCGA datasets. Log-rank test. (ADSSL1^low^ = 83; ADSSL1^high^ =84; IMPDH1^low^ = 83; IMPDH1^high^ =84).


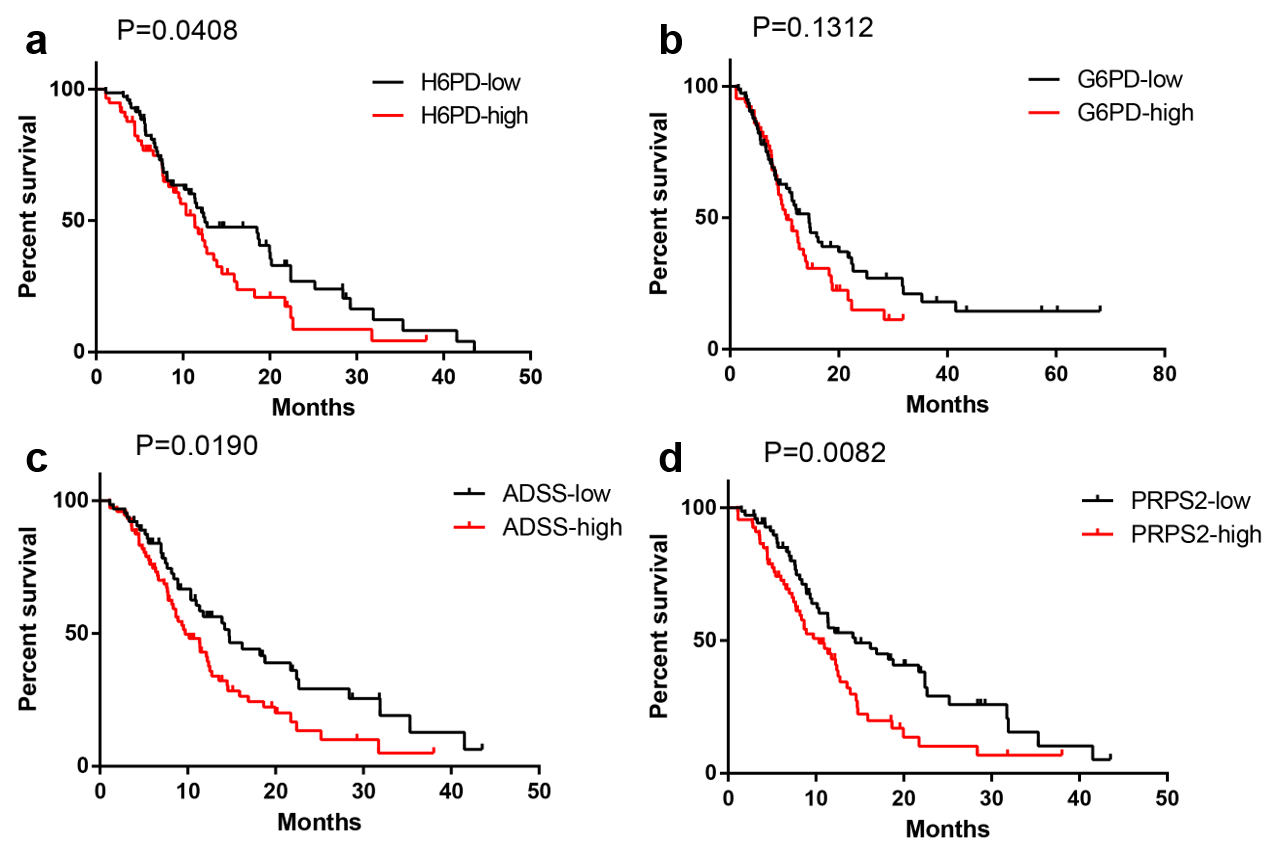


**Fig.S10** Analysis of glioblastoma patient survival based on H6PD, G6PD, ADSS and PRPS2 expression according to CGGA datasets. Log-rank test. (H6PD^low^ = 73; H6PD^high^ =65; G6PD^low^ = 73; G6PD^high^ =76; ADSS ^low^ = 63; ADSS^high^ =70; PRPS2^low^ = 70; PRPS2^high^ =66).

**2) SupplementaryTable.**

**Table S1** Parameters of the LC-MS/MS-based targeted metabolomic analysis of GSC2 in positive ion mode.

| **Q1 Mass (Da)** | **Q3 Mass (Da)** | **DP (V)** | **CE (eV)** | **Q1 Mass (Da)** | **Q3 Mass (Da)** | **DP (V)** | **CE (eV)** |
| --- | --- | --- | --- | --- | --- | --- | --- |
| 76.0 | 30.0 | 50 | 21 | 165.1 | 123.0 | 80 | 25 |
| 89.1 | 72.1 | 60 | 25 | 166.1 | 120.1 | 60 | 55 |
| 90.0 | 44.0 | 60 | 15 | 175.1 | 70.1 | 60 | 10 |
| 91.0 | 45.0 | 60 | 15 | 176.1 | 70.1 | 60 | 15 |
| 104.1 | 58.1 | 40 | 14 | 179.0 | 76.0 | 60 | 25 |
| 104.1 | 87.0 | 40 | 14 | 182.1 | 154.1 | 60 | 25 |
| 104.1 | 60.1 | 60 | 25 | 182.1 | 136.1 | 70 | 35 |
| 104.1 | 87.0 | 60 | 25 | 184.1 | 86.1 | 60 | 25 |
| 106.0 | 60.0 | 55 | 25 | 188.1 | 146.1 | 80 | 10 |
| 110.0 | 65.0 | 60 | 35 | 188.2 | 72.1 | 60 | 25 |
| 112.1 | 95.0 | 60 | 25 | 190.1 | 80.4 | 70 | 20 |
| 113.0 | 70.0 | 90 | 23 | 190.1 | 130.1 | 70 | 30 |
| 114.1 | 44.0 | 70 | 40 | 198.1 | 110.1 | 60 | 15 |
| 115.1 | 70.1 | 60 | 45 | 203.2 | 84.1 | 60 | 25 |
| 116.1 | 70.1 | 60 | 50 | 203.2 | 129.1 | 60 | 25 |
| 118.1 | 91.1 | 60 | 15 | 204.1 | 85.0 | 50 | 50 |
| 118.1 | 72.1 | 50 | 45 | 204.1 | 96.0 | 50 | 25 |
| 120.1 | 74.1 | 60 | 25 | 205.1 | 146.1 | 60 | 40 |
| 120.1 | 105.0 | 60 | 25 | 209.1 | 146.1 | 60 | 45 |
| 122.0 | 105.0 | 60 | 20 | 218.1 | 85.0 | 60 | 25 |
| 129.1 | 84.1 | 60 | 15 | 220.1 | 184.1 | 60 | 25 |
| 130.1 | 56.1 | 60 | 25 | 232.2 | 85.0 | 60 | 25 |
| 131.0 | 103.1 | 50 | 15 | 237.1 | 120.0 | 50 | 25 |
| 131.1 | 114.1 | 60 | 25 | 244.1 | 112.1 | 60 | 50 |
| 132.1 | 90.0 | 70 | 18 | 245.2 | 86.1 | 60 | 25 |
| 132.1 | 86.1 | 60 | 25 | 245.2 | 100.1 | 70 | 45 |
| 133.1 | 70.1 | 60 | 38 | 246.2 | 60.1 | 80 | 25 |
| 134.0 | 74.0 | 60 | 25 | 247.1 | 86.1 | 60 | 25 |
| 137.0 | 119.0 | 60 | 15 | 250.1 | 84.0 | 60 | 25 |
| 137.0 | 110.0 | 60 | 15 | 258.1 | 104.1 | 70 | 55 |
| 146.2 | 112.1 | 60 | 25 | 261.1 | 132.1 | 70 | 25 |
| 147.1 | 84.0 | 60 | 45 | 269.1 | 137.0 | 70 | 65 |
| 148.1 | 102.1 | 60 | 25 | 274.3 | 88.1 | 60 | 18 |
| 150.1 | 104.1 | 60 | 25 | 284.1 | 152.1 | 60 | 55 |
| 152.1 | 135.0 | 80 | 15 | 298.1 | 136.1 | 60 | 45 |
| 153.0 | 69.0 | 60 | 25 | 326.3 | 62.1 | 50 | 45 |

**Table S1** Continued.

| **Q1 Mass (Da)** | **Q3 Mass (Da)** | **DP (V)** | **CE (eV)** | **Q1 Mass (Da)** | **Q3 Mass (Da)** | **DP (V)** | **CE (eV)** |
| --- | --- | --- | --- | --- | --- | --- | --- |
| 153.0 | 108.0 | 60 | 35 | 308.1 | 76.0 | 60 | 10 |
| 156.1 | 95.1 | 60 | 40 | 348.1 | 136.1 | 60 | 10 |
| 162.1 | 85.0 | 70 | 15 | 364.1 | 152.1 | 60 | 25 |
| 372.3 | 85.0 | 80 | 45 | 508.3 | 367.3 | 60 | 25 |
| 398.3 | 85.0 | 80 | 45 | 516.3 | 104.1 | 60 | 25 |
| 400.3 | 85.0 | 80 | 15 | 520.3 | 104.1 | 60 | 15 |
| 426.4 | 85.0 | 80 | 45 | 520.3 | 184.1 | 60 | 15 |
| 428.4 | 85.0 | 50 | 45 | 522.4 | 184.1 | 60 | 10 |
| 435.2 | 205.1 | 60 | 40 | 524.4 | 184.1 | 60 | 25 |
| 452.3 | 311.3 | 60 | 25 | 550.4 | 184.1 | 50 | 45 |
| 454.3 | 313.2 | 60 | 25 | 568.3 | 184.1 | 80 | 35 |
| 466.3 | 325.3 | 60 | 25 | 664.1 | 136.1 | 80 | 40 |
| 468.3 | 184.1 | 80 | 45 | 666.1 | 302.0 | 80 | 40 |
| 478.3 | 337.3 | 60 | 35 | 210.0 | 192.0 | 60 | 25 |
| 480.3 | 339.3 | 60 | 45 | 414.3 | 378.3 | 80 | 10 |
| 482.3 | 184.1 | 50 | 45 | 401.0 | 85.0 | 80 | 15 |
| 494.3 | 184.1 | 60 | 25 | 192.0 | 145.0 | 60 | 20 |
| 496.3 | 184.1 | 60 | 10 | 117.1 | 71.1 | 55 | 5 |
| 502.3 | 361.3 | 60 | 25 |  |  |  |  |

**Table S2** The parameters of the LC-MS/MS-based targeted metabolomic analysis of GSC2 in negative ion mode.

| **Q1 Mass (Da)** | **Q3 Mass (Da)** | **DP (V)** | **CE (eV)** | **Q1 Mass (Da)** | **Q3 Mass (Da)** | **DP (V)** | **CE (eV)** |
| --- | --- | --- | --- | --- | --- | --- | --- |
| 89.0 | 43.0 | -70 | -20 | 450.3 | 253.2 | -70 | -15 |
| 108.0 | 64.0 | -50 | -10 | 452.3 | 255.2 | -70 | -20 |
| 124.0 | 80.0 | -70 | -25 | 464.3 | 267.2 | -70 | -20 |
| 147.0 | 129.0 | -70 | -12 | 474.3 | 277.2 | -70 | -15 |
| 147.0 | 103.1 | -70 | -15 | 476.3 | 279.2 | -70 | -15 |
| 151.1 | 71.0 | -95 | -24 | 478.3 | 281.2 | -70 | -15 |
| 155.0 | 68.0 | -50 | -25 | 480.3 | 283.3 | -70 | -20 |
| 155.0 | 111.0 | -50 | -17 | 483.0 | 385.0 | -70 | -15 |
| 160.1 | 142.0 | -70 | -20 | 483.3 | 255.2 | -70 | -35 |
| 160.1 | 116.1 | -70 | -20 | 498.3 | 301.2 | -70 | -20 |
| 164.1 | 147.0 | -70 | -20 | 500.3 | 303.2 | -70 | -50 |
| 167.0 | 124.0 | -70 | -20 | 506.0 | 408.0 | -70 | -15 |
| 179.1 | 89.0 | -90 | -13 | 507.3 | 279.2 | -80 | -50 |
| 179.1 | 87.0 | -70 | -21 | 509.3 | 281.2 | -70 | -40 |
| 181.1 | 59.0 | -100 | -15 | 522.3 | 153.0 | -70 | -30 |
| 188.1 | 102.0 | -80 | -20 | 526.3 | 329.2 | -70 | -20 |
| 188.1 | 128.0 | -80 | -20 | 526.4 | 466.3 | -70 | -20 |
| 191.0 | 111.0 | -70 | -37 | 540.1 | 79.0 | -80 | -50 |
| 199.2 | 45.0 | -70 | -40 | 540.3 | 255.2 | -70 | -40 |
| 218.1 | 88.0 | -70 | -20 | 546.3 | 281.0 | -70 | -60 |
| 243.1 | 110.0 | -70 | -20 | 548.3 | 480.3 | -80 | -40 |
| 267.1 | 135.0 | -70 | -60 | 565.0 | 323.0 | -70 | -35 |
| 271.1 | 203.1 | -70 | -20 | 566.3 | 224.1 | -70 | -30 |
| 282.1 | 150.0 | -70 | -20 | 571.3 | 255.2 | -80 | -45 |
| 283.1 | 151.0 | -70 | -20 | 595.3 | 279.2 | -70 | -40 |
| 306.1 | 210.1 | -70 | -20 | 597.3 | 281.2 | -70 | -40 |
| 323.0 | 211.0 | -90 | -20 | 606.1 | 385.0 | -70 | -40 |
| 346.1 | 211.0 | -70 | -20 | 611.1 | 306.1 | -70 | -20 |
| 347.0 | 79.0 | -70 | -15 | 662.1 | 540.1 | -70 | -20 |
| 362.1 | 79.0 | -70 | -20 | 742.1 | 620.0 | -70 | -15 |
| 363.0 | 211.0 | -70 | -25 | 208.0 | 120.1 | -110 | -24 |
| 388.9 | 158.9 | -80 | -20 | 121.0 | 76.0 | -40 | -10 |
| 388.9 | 291.0 | -70 | -25 | 412.3 | 350.3 | -70 | -15 |
| 426.0 | 328.0 | -70 | -20 | 190.0 | 127.0 | -100 | -25 |

**Table S3** Differential metabolites of GSC2 cultured under different pH conditions.

| **Metabolite identification** | ***m/z*** | **Adduct ion** | **Elemental composition** | **p** | **FC (6.8/7.4)** |
| --- | --- | --- | --- | --- | --- |
| XMP^a^ | 363.0346 | [M-H]- | C_10_H_13_N_4_O_9_P | 1.1E-27 | 18.2 |
| Uridine^a^ | 243.0616 | [M-H]- | C_9_H_12_N_2_O_6_ | 1.2E-17 | 5.8 |
| Uracil^a^ | 113.0349 | [M+H]+ | C_4_H_4_N_2_O_2_ | 3.0E-19 | 4.2 |
| Guanosine^a^ | 282.0840 | [M-H]- | C_10_H_13_N_5_O_5_ | 3.9E-11 | 3.9 |
| Carnitine (C5:0) ^b^ | 246.1702 | [M+H]+ | C_12_H_23_NO_4_ | 2.3E-24 | 3.7 |
| AMP^a^ | 348.0699 | [M+H]+ | C_10_H_14_N_5_O_7_P | 4.5E-24 | 3.4 |
| GMP^a^ | 362.0503 | [M-H]- | C_10_H_14_N_5_O_8_P | 4.2E-26 | 3.4 |
| 8-Hydroxy-7-methylguanine^b^ | 182.0672 | [M+H]+ | C_6_H_7_N_5_O_2_ | 3.4E-11 | 3.3 |
| Phosphocholine^a^ | 184.0730 | [M+H]+ | C_5_H_14_NO_4_P | 2.8E-29 | 3.2 |
| gamma-Glu-cys^b^ | 251.0696 | [M+H]+ | C_8_H_14_N_2_O_5_S | 3.0E-12 | 3.1 |
| UDP-N-acetylglucosamine^a^ | 606.0738 | [M-H]- | C_17_H_27_N_3_O_17_P_2_ | 2.2E-24 | 3.0 |
| NADP^a^ | 742.0684 | [M-H]- | C_21_H_29_N_7_O_17_P_3_ | 2.4E-19 | 2.8 |
| Inosine^a^ | 267.0732 | [M-H]- | C_10_H_12_N_4_O_5_ | 6.2E-08 | 2.8 |
| UMP^a^ | 323.0279 | [M-H]- | C_9_H_13_N_2_O_9_P | 6.1E-05 | 2.5 |
| L-Glutamate^a^ | 148.0604 | [M+H]+ | C_5_H_9_NO_4_ | 2.8E-24 | 2.5 |
| Choline^a^ | 104.1074 | [M+H]+ | C_5_H_13_NO | 5.3E-23 | 2.5 |
| 2-Hydroxyglutaric acid^a^ | 147.0285 | [M-H]- | C_5_H_8_O_5_ | 7.5E-25 | 2.4 |
| GABA^a^ | 104.0710 | [M+H]+ | C_4_H_9_NO_2_ | 5.2E-21 | 2.3 |
| UDP-glucose^b^ | 565.0477 | [M-H]- | C_15_H_24_N_2_O_17_P_2_ | 2.9E-28 | 2.2 |
| Cytosine^a^ | 112.0509 | [M+H]+ | C_4_H_5_N_3_O | 1.3E-10 | 2.2 |
| Citric acid^a^ | 191.0184 | [M-H]- | C_6_H_8_O_7_ | 1.1E-16 | 2.1 |
| Cytidine ^a^ | 244.0927 | [M+H]+ | C_9_H_13_N_3_O_5_ | 2.0E-21 | 2.1 |
| Reduced glutathione^a^ | 306.0762 | [M-H]- | C_10_H_17_N_3_O_6_S | 1.6E-15 | 2.0 |
| Hypoxanthine^a^ | 137.0459 | [M+H]+ | C_5_H_4_N_4_O | 1.2E-12 | 2.0 |
| N-Acetyl-D-glucosamine^b^ | 204.0865 | [M+H-H2O]+ | C8H15NO6 | 8.2E-15 | 2.0 |
| L-Glutamine^a^ | 147.0764 | [M+H]+ | C_5_H_10_N_2_O_3_ | 4.9E-19 | 2.0 |
| Cys-gly^b^ | 179.0485 | [M+H]+ | C_5_H_10_N_2_O_3_S | 1.9E-15 | 2.0 |
| L-Lysine^a^ | 129.1023 | [M+H-H2O]+ | C_6_H_14_N_2_O_2_ | 6.8E-24 | 1.9 |
| Carnitine (C4:0)^b^ | 232.1544 | [M+H]+ | C_11_H_21_NO_4_ | 8.0E-15 | 1.9 |
| ATP^a^ | 505.9887 | [M-H]- | C_10_H_16_N_5_O_13_P_3_ | 7.8E-12 | 1.9 |
| IMP^a^ | 347.0394 | [M-H]- | C_10_H_13_N_4_O_8_P | 3.0E-17 | 1.9 |
| LysoPE (18:3)^b^ | 474.2618 | [M-H]- | C_23_H_42_NO_7_P | 8.8E-08 | 1.9 |
| LysoPC (18:2)^b^ | 520.3397 | [M+H]+ | C_26_H_50_NO_7_P | 7.5E-03 | 1.8 |
| N-Acetyl-L-histidine^b^ | 222.0953 | [M+H]+ | C_8_H_15_NO_6_ | 1.2E-08 | 1.7 |
| Xanthine^a^ | 153.0408 | [M+H]+ | C_5_H_4_N_4_O_2_ | 1.4E-12 | 1.6 |
| LysoPE (18:2)^b^ | 476.2777 | [M-H]- | C_23_H_44_NO_7_P | 1.4E-06 | 1.5 |
| L-Arginine^a^ | 175.1189 | [M+H]+ | C_6_H_14_N_4_O_2_ | 2.6E-14 | 1.4 |
| PG (16:0)^b^ | 483.2721 | [M-H]- | C_22_H_45_O_9_P | 7.0E-04 | 1.4 |
| L-Formylkynurenine^a^ | 237.0868 | [M+H]+ | C_11_H_12_N_2_O_4_ | 6.1E-06 | 1.3 |

**Table S3** Continued.

| **Metabolite identification** | ***m/z*** | **Adduct ion** | **Elemental composition** | **p** | **FC (6.8/7.4)** |
| --- | --- | --- | --- | --- | --- |
| Spermidine^a^ | 146.1652 | [M+H]+ | C_7_H_19_N_3_ | 2.1E-11 | 1.3 |
| L-Carnitine^a^ | 162.1124 | [M+H]+ | C_7_H_15_NO_3_ | 6.6E-03 | 1.3 |
| L-Prolinamide^b^ | 115.0870 | [M+H]+ | C_5_H_10_N_2_O | 2.0E-01 | 1.0 |
| Oxidized glutathione^a^ | 611.1444 | [M-H]- | C_20_H_32_N_6_O_12_S_2_ | 2.7E-01 | 0.9 |
| LysoPE (16:0)^b^ | 454.2925 | [M+H]+ | C_21_H_44_NO_7_P | 3.3E-05 | 0.8 |
| LysoPC (15:0)^b^ | 482.3241 | [M+H]+ | C_23_H_48_NO_7_P | 1.7E-03 | 0.7 |
| Carnitine (C14:0)^b^ | 372.3108 | [M+H]+ | C_21_H_41_NO_4_ | 2.2E-09 | 0.5 |
| Cystamine^b^ | 153.0515 | [M+H]+ | C_4_H_12_N_2_S_2_ | 4.1E-11 | 0.4 |
| Carnitine (C18:1)^b^ | 426.3578 | [M+H]+ | C_25_H_47_NO_4_ | 1.1E-12 | 0.4 |
| 5-Methylthioadenosine^a^ | 298.0968 | [M+H]+ | C_11_H_15_N_5_O_3_S | 1.1E-05 | 0.3 |
| Creatinine^a^ | 114.0662 | [M+H]+ | C_4_H_7_N_3_O | 8.8E-25 | 0.3 |
| Carnitine (C16:1)^b^ | 398.3265 | [M+H]+ | C_23_H_43_NO_4_ | 1.2E-16 | 0.3 |

^a^ Metabolites were confirmed using standard compounds. ^b^ Metabolites were identified by database searches and MS fragmentation.
